# The Role of Grain Boundary Sites for the Oxidation of Copper Catalysts during the CO Oxidation Reaction

**DOI:** 10.1021/acsnano.3c06282

**Published:** 2023-10-05

**Authors:** Sara Nilsson, John N. El Berch, David Albinsson, Joachim Fritzsche, Giannis Mpourmpakis, Christoph Langhammer

**Affiliations:** † Department of Physics, Chalmers University of Technology, 412 96 Göteborg, Sweden; ‡ Department of Chemical and Petroleum Engineering, University of Pittsburgh, Pittsburgh, Pennsylvania 15261, United States

**Keywords:** grain boundary sites, CO oxidation, surface oxidation, single particle, plasmonic nanoimaging, DFT, copper nanoparticles

## Abstract

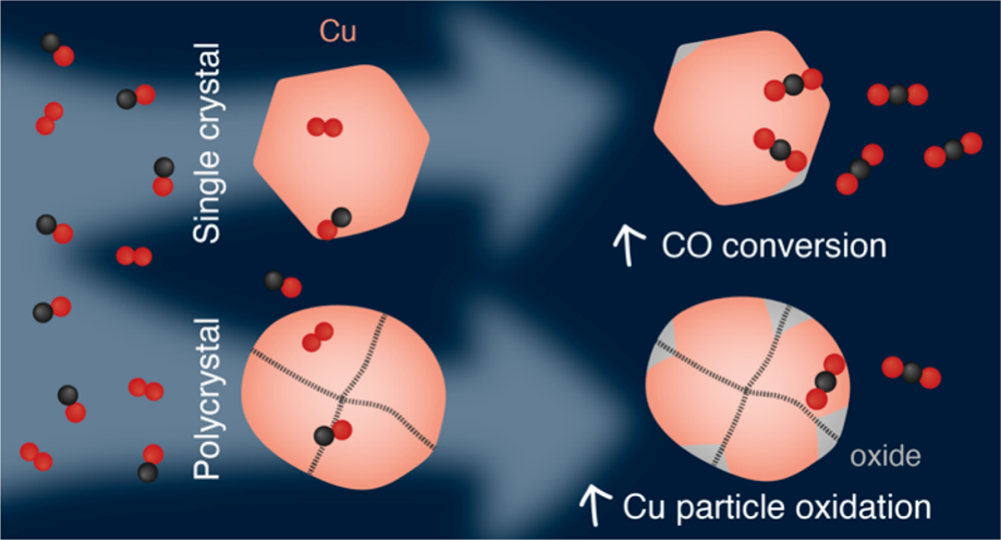

The oxidation of transition metal surfaces is a process that takes place readily at ambient conditions and that, depending on the specific catalytic reaction at hand, can either boost or hamper activity and selectivity. Cu catalysts are no exception in this respect since they exhibit different oxidation states for which contradicting activities have been reported, as, for example, in the catalytic oxidation of CO. Here, we investigate the impact of low-coordination sites on nanofabricated Cu nanoparticles with engineered grain boundaries on the oxidation of the Cu surface under CO oxidation reaction conditions. Combining multiplexed *in situ* single particle plasmonic nanoimaging, *ex situ* transmission electron microscopy imaging, and density functional theory calculations reveals a distinct dependence of particle oxidation rate on grain boundary density. Additionally, we found that the oxide predominantly nucleates at grain boundary-surface intersections, which leads to nonuniform oxide growth that suppresses Kirkendall-void formation. The oxide nucleation rate on Cu metal catalysts was revealed to be an interplay of surface coordination and CO oxidation behavior, with low coordination favoring Cu oxidation and high coordination favoring CO oxidation. These findings explain the observed single particle-specific onset of Cu oxidation as being the consequence of the individual particle grain structure and provide an explanation for widely distributed activity states of particles in catalyst bed ensembles.

## Introduction

Due to the scarcity of many transition metals widely employed as catalysts, such as Pt, Pd, and Rh, there is a need for more abundant metals for catalysis applications. In this context, Cu is an interesting catalyst since it exhibits high activity toward several industrially relevant reactions, such as the oxidation of CO at low temperatures,^1^ the water gas shift reaction,^2^ and the methanol synthesis based on Cu/ZnO/Al_2_O_3_ catalysts.^3^ Despite its diverse applications in catalysis, there is still debate about the active phase of Cu in different reactions since it readily oxidizes into various oxidation states.^4^ Taking the CO oxidation reaction as an example, metallic Cu,^5−7^ Cu_2_O,^8,9^ and CuO^10^ have all been reported to be active phases. It is also worth noting that the specific reaction conditions reported in the literature often vary significantly, which, alongside the surface sensitivity of the experimental techniques, are likely the reasons for the different conclusions about the active phase. Moreover, it is probable that there is no single active phase, but rather a dynamic interplay between (surface) oxides and metallic surfaces that determines the catalytic activity at the atomic level.^11^ To this end, high-pressure scanning tunneling microscopy (HP-STM)^12^ and environmental transmission electron microscopy (ETEM)^13^ have, with high spatial resolution, provided evidence of Cu surface restructuring and Cu adatom clustering upon exposure to CO, where step edges are the first surface sites to reconstruct.^12^ Translating this information mostly obtained on stepped (single crystalline) surfaces onto more practically relevant systems of nanoparticles reveals structure sensitivity, as demonstrated for colloidal Cu_2_O nanoparticles.^14^ Similarly, grain boundaries, which constitute areas of high defect density with an abundance of low-coordinated sites, have been demonstrated to be important in Cu surface oxidation,^15,16^ in hydrogen sorption on Pd nanoparticles^17^ and in electrocatalysis.^18−20^ Hence, it is not far-fetched to anticipate that grain boundaries play a significant role also in the CO oxidation reaction.

Herein, we study the oxidation of up to 225 electron-beam lithographically (EBL) fabricated and thermally annealed Cu nanoparticles in one sample, with grain morphologies ranging from single crystals to polycrystals with up to 10 grains. We use this wide range of grain morphologies as a model for emulating the role of atomic sites with different coordination environments exposed to CO oxidation conditions. By pairing *in situ* plasmonic nanoimaging^6,21^ and *ex situ* annular dark-field scanning transmission electron microscopy (ADF-STEM) with atomic insights from density functional theory (DFT) calculations, we connect the observed earlier onset of subsurface oxide nucleation in the polycrystals to the high abundance of low-coordinated sites located at the grain boundaries. Furthermore, we engineer samples hosting different grain boundary distributions by varying the pretreatment temperature and observe a nonlinear dependence of the oxidation onset on the annealing temperature, which stems from the interplay between strong CO adsorption on low-coordinated sites and high CO oxidation activity at high-coordinated sites.

## Results

### Experimental Sample Preparation and Characterization

**Figure 1:**
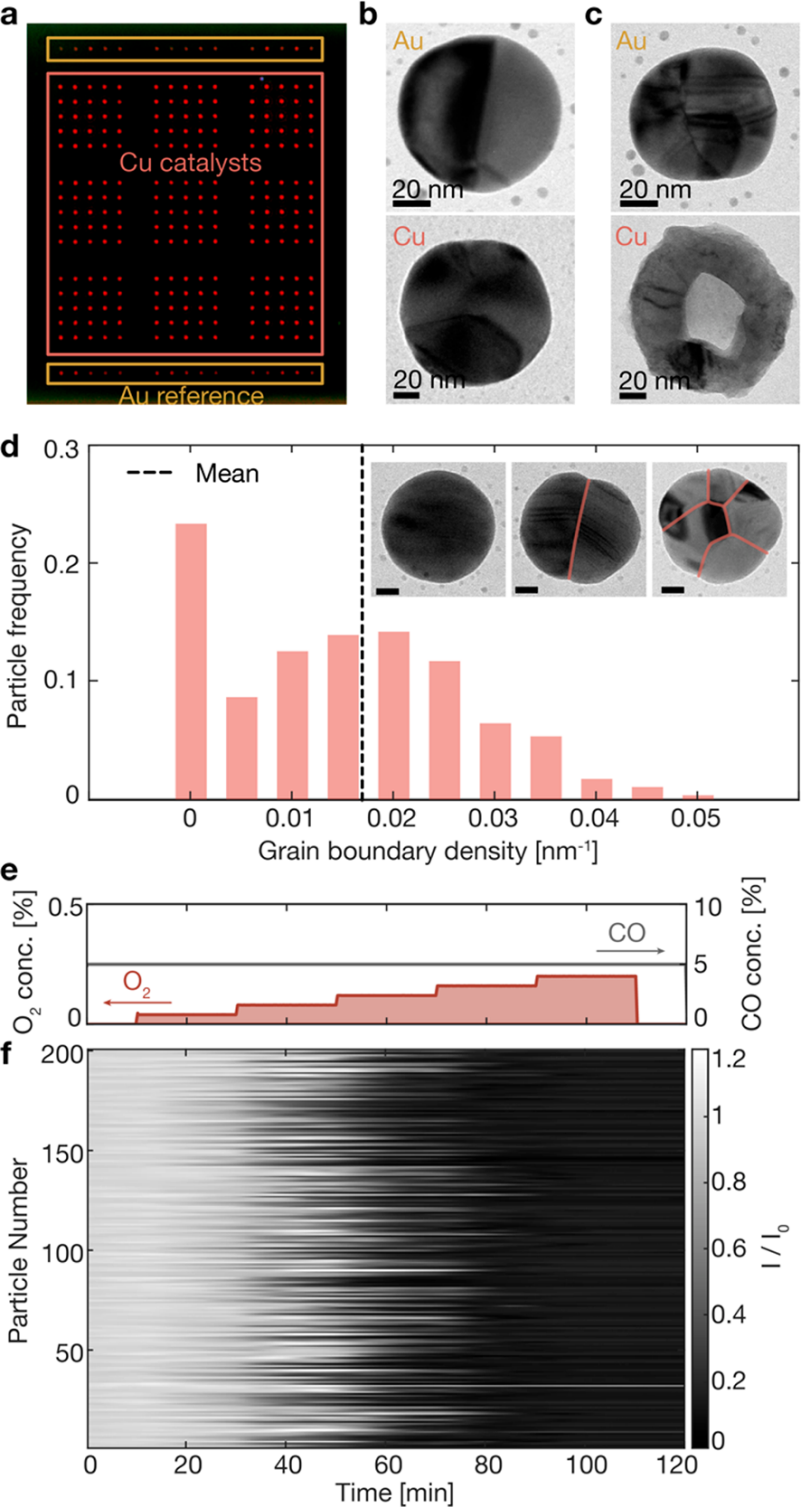
Optical nanoimaging of Cu nanoparticles with wide grain boundary density distribution. (a) A dark-field scattering microscope color image of a thermally annealed particle array imaged in a stream of 2% H_2_ in Ar before starting exposure to CO and O_2_. The array is comprised of 225 Cu particles in the center and 30 oxidation-resistant, optical reference Au particles at the top and bottom (alternative sample design in Figure S1.1 with 200 Cu and 25 Au nanoparticles). Bright-field TEM images of one representative Au (top) and Cu particle (bottom) (b) taken prior to exposure to reaction conditions and (c) two other particles after 100 min of exposure to reaction conditions according to (e). Clearly, the Au particle is not oxidized (intensity traces in Figure S1.2) whereas the Cu particle is completely oxidized and has formed a void in its center. All scale bars are 20 nm. (d) Histogram of the grain boundary density derived from TEM images of 878 nanoparticles annealed in 2% H_2_ in Ar at 400 °C. The mean and standard deviation of the grain boundary density distribution is 0.017 ± 0.013 nm^–1^. Inset: TEM images of three representative Cu particles, one single crystal and two polycrystals, showing the diversity in grain morphology engineered in one sample. All scale bars are 20 nm. (e) The CO oxidation reaction gas mixture with increasing O_2_ concentration from 0% to 0.2% in steps of 0.04 in a constant background of 5% CO in Ar carrier gas at atmospheric pressure and at 250 °C. (f) Normalized scattering intensity profiles recorded simultaneously for 200 single Cu nanoparticles under the reaction conditions depicted in (e). Note that the transition from a bright to a dark state appears seemingly random for the individual particles with no dependence on the particle position in the array.

We prepared EBL-fabricated, disk-like Cu particles of 110 nm nominal diameter and 40 nm height in regular arrays with a 4 μm particle–particle distance onto transmission electron microscopy (TEM) “windows” comprised of a 25 nm thin SiN_*x*_ membrane.^22^ Furthermore, we included an array of Au nanoparticles (diameter 110 nm and height 20 nm), which serve as an oxidation-resistant optical reference during the plasmonic nanoimaging measurements to account for, e.g., intensity fluctuations of the used light source and the overall background scattering (Figure 1a–c, S1.1–1.2). In this way, the time evolution of the scattering intensity, *I*, for each Cu particle can be monitored at identical reaction conditions. Using this concept, we have previously observed that, in a flow of pure O_2_ in Ar carrier gas, oxide formation both spectrally shifts and reduces the localized surface plasmon resonance (LSPR) signature of the particles proportionally to the amount of oxide formed.^23^

To control the grain boundary density of the Cu particles in the array, and to obtain a range of different grain morphologies, we annealed the samples at 400 °C in 2% H_2_ in Ar carrier gas prior to reaction conditions (see Methods Section for details). This thermal pretreatment yielded a distribution of particle grain morphologies within one sample (Figure 1d). In the analysis of the role of these grain boundaries on the Cu particle oxidation process under CO oxidation reaction conditions, from here onward, we group the particles prepared in this way into two classes: (i) single crystals, without any grain boundaries, and (ii) polycrystals, containing two or more grains (examples in the inset of Figure 1d). To this end, we have previously compared our methodology (see Methods Section for details) of measuring the grain boundary length in particles from TEM images with the analysis from transmission Kikuchi diffraction (TKD) of the same particles and found a median error of 23% when comparing 78 Cu particles.^21^ This means that our method allows us to estimate the grain boundary length from TEM images to below 23% error in half of the particles. We should also consider that TKD has some uncertainty in measuring the grain boundary length, e.g., underestimating it by not recognizing small grains.

For the oxidation experiments, we implemented the reaction conditions as a constant CO background concentration of 5% in Ar carrier gas at atmospheric pressure and by increasing the O_2_ concentration from 0% in steps of 0.04 percent up to 0.2% (Figure 1e). Simultaneously, we extracted the time evolution of the optical signature of each single Cu particle, *I*/*I*_0_, where *I*_0_ is the scattering intensity of each particle at the beginning of the experiment in 5% CO without the presence of O_2_ (Figure 1f).

This analysis reveals a large spread in the onset time of Cu particle oxidation along the time coordinate, marked as a distinct transition from an optically bright to an optically dark state for the individual nanoparticles, that extends over a time window of more than 30 min and three distinct O_2_ concentration steps. As a first observation, the transition seems to appear randomly with no dependence on particle position in the array. This implies a highly particle-specific affinity to oxidation of the Cu particle itself during the CO oxidation reaction, in good agreement with our past observations.^6^ We also note that under the CO oxidation reaction conditions applied here, a linear correlation (with Pearson coefficient −0.62) between the optical *I*/*I*_0_ signature of each particle and its oxidation fraction can be established up to around 40% volume oxidation, at which point the light scattered from the small remaining metallic volume is very weak. At the same time, there is a small but increasing scattering contribution from the growing oxide, which explains why we do not observe a further decrease in scattering intensity (SI Section S2). This means that in analogy to our earlier results obtained for oxidation from O_2_ in Ar,^23^ the measured change in light scattering intensity for each particle is proportional to its oxidation fraction up until 40% volume oxidation.

### Comparing Oxide Growth on Single- and Polycrystalline Cu Particles under CO Oxidation Reaction Conditions

**Figure 2:**
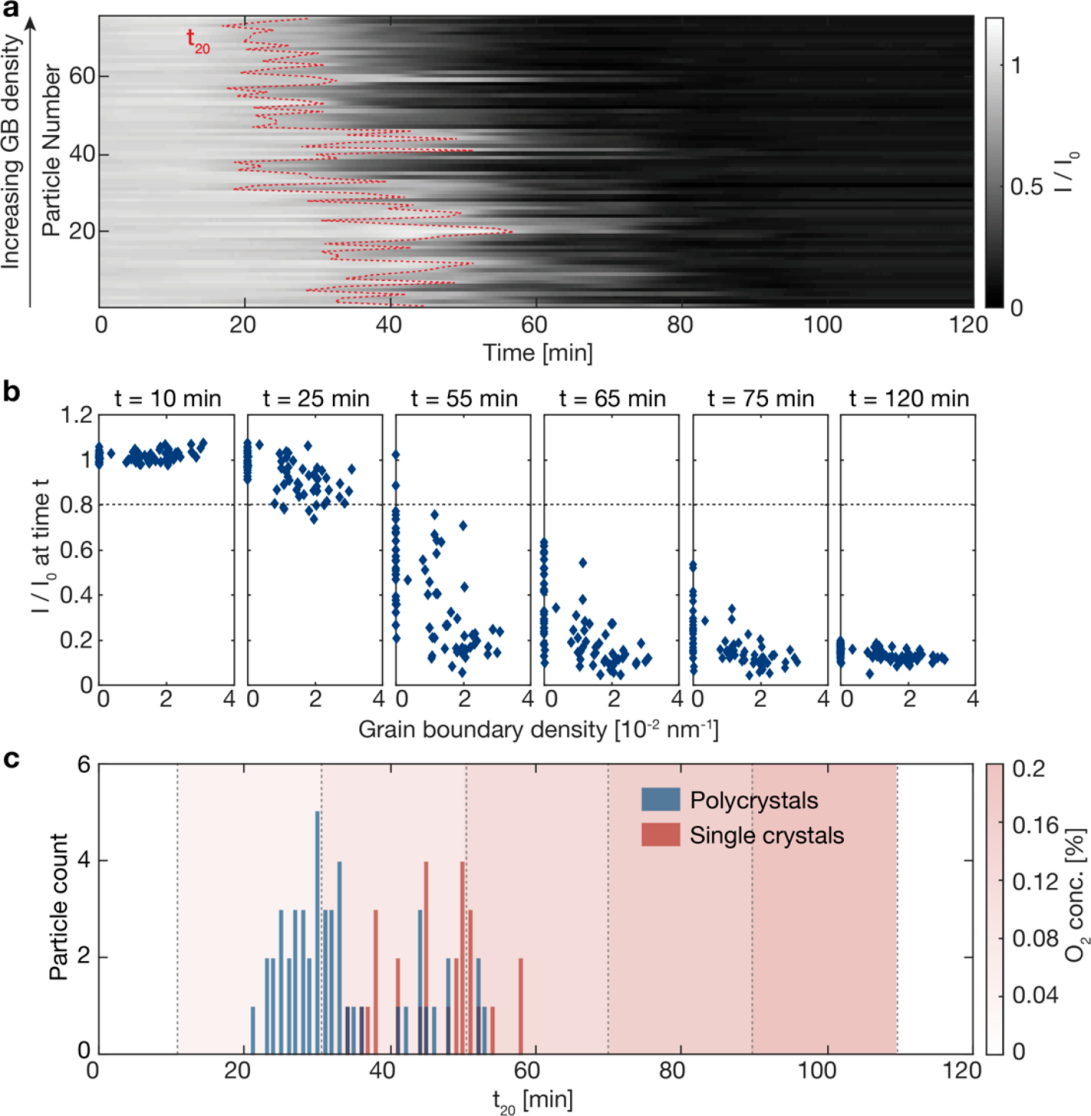
*In situ* single particle light scattering intensity readout. (a) Normalized scattering intensity traces, *I*/*I*_0_, of 75 Cu particles imaged with bright-field TEM prior to exposure to reaction conditions to characterize their grain morphology. Note that these data are a subset of the 200-particle set depicted in Figure 1f, and that the particles are sorted on the *y*-axis according to their grain boundary density determined from the TEM images. The dashed line depicts *t*_20_ for each particle, the point along the time axis at which *I* is reduced by 20% from its initial value, *I*_0_. Note that particles with high grain boundary density transit to a darker, oxidized state earlier. (b) Relative scattering intensities *I*/*I*_0_ for all 75 particles extracted after 10, 25, 55, 65, 75, and 120 min along the reaction sequence (cf. Figure 1e, O_2_ exposure starts after 10 min) and plotted as a function of the grain boundary density derived from the TEM images taken prior to this exposure. (c) Distribution of *t*_20_ for the 75 particles in (a, b) grouped into single crystals (red) and polycrystals (blue). The red fields mark the O_2_ gas concentration during the reaction, i.e., 0.04, 0.08, 0.12, 0.16, and 0.2%. For the single crystals, the extracted *t*_20_ values have mean and standard deviation *t̅*_20_ = 47 ± 6.6 min and for the polycrystals *t̅*_20_ = 34 ± 8.5 min.

For the next step in scrutinizing the dependence of the time stamp of Cu particle oxidation onset on their grain morphology, we imaged 75 out of the 200 particles in the array by *ex situ* bright-field TEM *prior* to the reaction experiment. Imaging 75 particles was chosen as the best trade-off between time invested in TEM imaging on each sample and the statistical relevance of the obtained data set. This allowed us to determine their grain boundary density in the pristine state and, during the data analysis, rearrange their scattering intensity traces from Figure 1f according to their respective grain boundary density (Figure 2a). To compare oxidation onset between particles, we define and extract *t*_20_, i.e., the time when the relative light scattering intensity, *I*/*I*_0_, from each of the 75 particles imaged by TEM has decreased by 20% compared to its initial value, *I*_0_. We choose 20% intensity decrease to ensure that the signal is safely above inherent fluctuations in the scattered light intensity that are not related to particle oxidation, as corresponding control experiments have revealed (Figure S3.6). In Figure 2a, *t*_20_ is marked by the red dashed line. Reorganizing the scattering intensity signatures accordingly reveals that, on average, particles with higher grain boundary densities experience an earlier onset of scattering intensity decrease, and thus start oxidizing earlier than single crystals or polycrystals with few grains (Figure 2a, Figure S3.2). Furthermore, extracting the *I*/*I*_0_ values for all 75 particles after 10, 25, 55, 65, 75, and 120 min, and plotting them as a function of their grain boundary density derived from the TEM images, reveals even more clearly both the earlier oxidation of particles with high grain boundary densities and the significant spread between individuals in terms of their oxide growth onset (Figure 2b). The delayed oxidation onset in single crystals compared to polycrystals also becomes evident when comparing the *t*_20_-distributions for these two particle classes (Figure 2c, additional data in Figure S3.7). We see that the oxidation onset in the polycrystals (blue bars) is close to a normal distribution with a mean and standard deviation of *t̅*_20_ = 34 ± 8.5 min, whereas for the single crystals (red bars) the oxidation onset is significantly delayed for the majority of particles to between 40 and 60 min, leading to a corresponding mean *t̅*_20_ = 47 ± 6.6 min.

**Figure 3:**
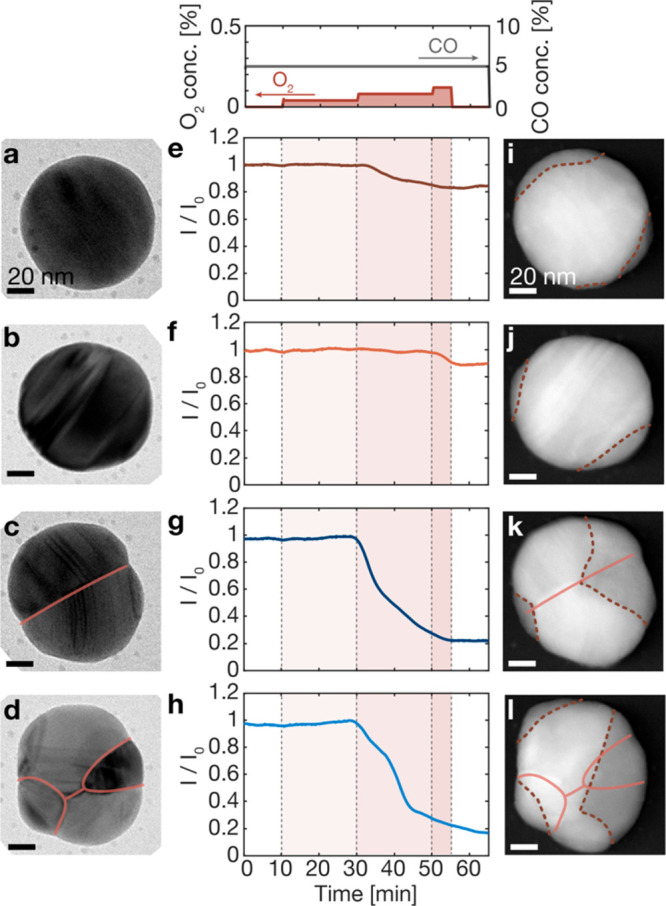
Examples of single and polycrystalline Cu nanoparticles exposed to CO oxidation reaction conditions. Bright-field TEM images of (a, b) two Cu single crystals and (c, d) two polycrystals acquired prior to exposure to reaction conditions. (e–h) Temporal evolution of their relative light scattering intensity, *I*/*I*_0_, during exposure to reaction conditions, as obtained by plasmonic nanoimaging. (i–l) ADF-STEM images of the same particles after exposure to the reaction gas mixture according to the panel on top of (e), revealing their different amount of oxidation, in good agreement with the scattering intensity decrease seen in (e–h). In both bright-field TEM and ADF-STEM images, the grain boundaries are highlighted by solid red lines, and in the ADF-STEM images, the interface between oxide and metal is highlighted by dashed lines. Scale bars are 20 nm.

To further elucidate any potential structural consequences of the apparent earlier oxidation onset of polycrystalline Cu particles, we performed a similar experiment to that depicted in Figure 1e. However, this time we interrupted the exposure to reaction conditions after 55 min for STEM imaging, since at this specific time we observed a large spread in the scattering intensities of the individual particles (cf. third panel of Figure 2b), which we hypothesize is because the polycrystals were more oxidized than the single crystals (scattering intensities of all 225 particles in the sample in Figure S3.8). From this experiment, we make two relevant observations. First, by comparing the bright-field TEM images of selected particles taken prior to their exposure to reaction conditions (Figure 3a–d), and ADF-STEM images of the same particles taken after 55 min in reaction conditions (Figure 3i–l), we observe a higher volume oxidation fraction for the two polycrystalline particles compared to the two single crystals. Second, when turning to analyze the optical response of the four example particles (Figure 3e–h), we can again see the trend observed in Figure 2a, namely that the scattering intensity change reflects the volume oxidation fraction and that the single crystalline particles are less oxidized than the polycrystalline ones. Notably, the scattering intensity change measured from the single crystals is smaller than 20% of *I*_0_ (Figure 3e,f), which means that these particles have not yet reached *t*_20_. This implies only shallow oxide growth. By image analysis, we measured the metal area, which we distinguished from the oxide area by the contrast difference, before and after the exposure to reaction conditions (characterization of the oxide in Figure S5.1). We have previously characterized the oxide by ADF-STEM and electron energy loss spectroscopy (EELS) to motivate the relevance of using the contrast difference in ADF-STEM images of similar particles.^24^ The image analysis reveals limited oxidation fractions in the two single crystals of about 9% (Figure 3i) and 3% (Figure 3j). In contrast, the two polycrystals display a significant intensity decrease by 80% of *I*_0_ (Figure 3g,h), which from measuring the oxidation fractions corresponds to about 25% (Figure 3k) and 35% (Figure 3l). Here, we reiterate that the scattering intensity decreases linearly with the oxidation fraction until about 40% of oxidation, at which point the scattering from the remaining metallic volume is too weak to be detected in the experiment (Figure S2.1).

As the next step, we study the ADF-STEM images taken after exposure to reaction conditions in more detail (Figure 3i–l). Starting with the single crystals, the formed oxide is nonuniformly distributed over a finite number of locations on the particle’s surface. This strongly contrasts the oxide formation in pure oxygen on similar^21,23−25^ and other types^4,27−29^ of Cu nanoparticles, for which homogeneous oxide shell growth has been reported. Focusing on the polycrystals, we make two important observations. The first one is that the polycrystalline particles indeed are significantly more oxidized than the single crystals, again in good agreement with the in situ plasmonic nanoimaging readout. We hypothesize that this is because a higher density of surface steps and other low coordination sites accompanied by lattice strain can be found at the intersection between the grain boundaries and the particle surface. Such sites have previously been reported to enhance the oxide formation rate^30^ and can act as channels for oxygen diffusion on the surface.^31^

The second observation is that the oxidation has progressed deeper into the polycrystalline particles than into the single crystals and is deepest at a position of a grain boundary, where typically an oxide apex has formed (Figure 3k, S4.1). This characteristic apex shape of the oxide, observed in many of the polycrystalline particles (further examples in Figure S8.1–8.2), can be explained by enhanced oxygen diffusion along grain boundaries^32^ and at atomic steps,^31^ which leads to a higher oxide growth rate along that boundary. Hence, from the surface, the oxide grows both inward along the boundary and sideways across the crystal lattice, therefore readily forming the wedge shape observed in the ADF-STEM images in our experiments. We have characterized the oxide by high resolution ADF-STEM and found that the lattice plane spacing agrees with Cu_2_O (SI Section S5), which we have also previously found by *ex situ* XPS after CO oxidation on similar particles at 400 °C.^6^

Our final observation is that, despite 25–35% of volume oxidation in the two polycrystals (Figure 3k,l) and 17 other polycrystals in the same sample, no Kirkendall voids are formed neither in the single crystals nor in the polycrystals (Figure S8.5–8.6). This observation distinctly contrasts earlier work on Cu nanoparticle oxidation in pure oxygen at higher (partial) pressures, where Kirkendall voids have been observed to form at 20–30% volume oxidation in similar kinds of particles.^23,24,28^ At the same time, Cu nanoparticles exposed to pure oxygen at lower pressures have been observed to oxidize without Kirkendall void formation.^33^ Hence, the observed absence of Kirkendall void formation during Cu nanoparticle oxidation here is likely the consequence of both slightly lower O_2_ partial pressure than in other studies, and the presence of CO (Figure 3i–l, Figure S9.2b). In a control experiment of oxidation in up to 1.2 mbar partial pressure O_2_ in Ar, we observe more but not completely homogeneous oxide growth (Figure S9.1, S9.2a). However, in the presence of CO, which both consumes O atoms for its oxidation to CO_2_ and adsorbs to the surface where it blocks some Cu sites from being oxidized, the Cu oxide growth is less homogeneously distributed and takes place only at a few positions on the particle. Hence, the difference from the cases where the Kirkendall void forms can mechanistically be understood as the consequence of the nucleation of oxide islands at only a couple of sites. From these few nucleation sites, the oxide continues to grow rather than nucleating further oxide islands, due to fast oxygen diffusion along surface steps.^31^ This contrasts the scenario observed in pure O_2_ at higher (partial) pressures, where a homogeneous oxide shell is formed by the coalescence of oxide islands that had nucleated simultaneously at a multitude of sites all over the particle surface.^23,24^ This spatially inhomogeneous oxide island nucleation and growth in the presence of CO thus prevents the formation of a completely enclosing oxide shell, which is the prerequisite for the diffusion rate contrast between oxygen and Cu ions across the oxide that is required to induce the formation and growth of Kirkendall voids.^4^

### Understanding the Impact of CO on Cu Oxide Nucleation and Growth

As introduced above, the oxidation of Cu surfaces in purely oxidating environments has been studied extensively.^34^ Initially, stable periodical oxygen overlayers are formed,^35^ such as the missing-row reconstruct (MRR), where every fourth Cu atom is missing, or the c(2 × 2) overlayer.^36^ Upon extended oxygen exposure, the stable overlayer is breached by Cu_2_O islands that nucleate on the surface and grow both horizontally and vertically, which leads to subsurface oxidation.^16,37^ The transition from MRR is suggested to take place after a critical oxygen exposure.^38^ Notably, however, grain boundaries, facet-edges and -corners, and step-edges can decrease the required oxygen exposure and facilitate oxide island nucleation.^16,30,39^ Apart from the mentioned studies on extended surfaces, we have also reported the oxide island growth by *in situ* ADF-STEM on similar Cu nanoparticles as investigated here.^24^

Translating the above to our experimental conditions, it becomes clear that starting from a CO-covered surface and sequentially exposing it to a CO + O_2_ reaction gas mixture constitutes significantly different conditions, for which the pathway of the subsurface Cu oxidation has not been as extensively studied. It is known that introducing CO roughens the Cu surface by creating clusters of Cu atoms on terraces that expose more low-coordinated surface atoms, which increases the number of steps and kinks.^12,13^ Furthermore, oxide islands present on the surface turn amorphous when exposed to CO, as recently shown by *in situ* TEM.^13^

**Figure 4:**
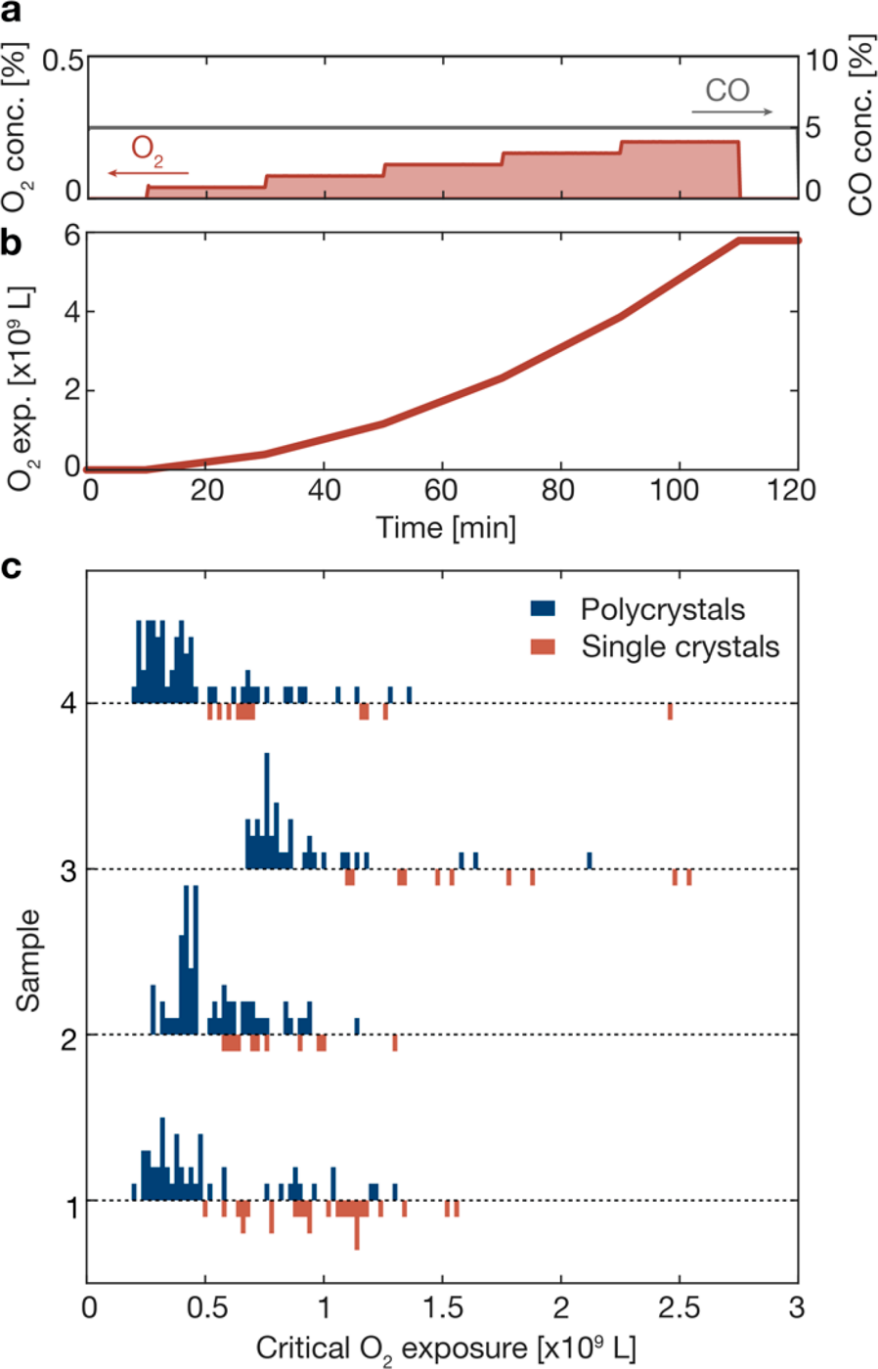
Grain boundaries facilitate Cu oxide nucleation under CO oxidation reaction conditions. (a) The reaction gas sequence (same as in Figure 1e). (b) The total oxygen exposure of the particles during the reaction sequence. Here, we do not consider the local consumption of O_2_ in oxidizing CO to CO_2_. (c) The critical O_2_ exposure of 59 single crystals (red) and 219 polycrystals (blue) from 4 samples annealed at 400 °C in 2% H_2_ (annealing times sample 1:4 h, sample 2:1 h and sample 3 and 4:2 h). The histogram bar heights are comparable between the samples. Note that in all samples the polycrystals start subsurface oxide formation earlier than single crystals.

To identify general trends and inspired by the reported critical O_2_ exposure necessary for subsurface oxidation in pure O_2_,^38^ we calculated the critical O_2_ exposure based on the *t*_20_ oxidation onset values previously determined for a large number of particles (cf. Figure 2, scattering intensities from all particles in Figure S3.1–3.5). The total O_2_ exposure was found by integrating the O_2_ flow during the experiment. Hence, extracting the O_2_ exposure at *t*_20_ for each particle provides a measure to compare how much O_2_ exposure each particle tolerates before it oxidizes appreciably (O_2_ exposure in Langmuir, 1 L = 10^–6^ Torr s, Figure 4a,b), i.e., each particle’s critical O_2_ exposure limit. In this analysis, we are not considering the simultaneous conversion of O_2_ with CO to CO_2_. We use a batch-type reactor and large particle–particle distances; therefore, interparticle effects are negligible, and the gas volume relative to the catalyst surface area is large. Furthermore, we can compare the O_2_ consumption with that of large-area samples (having catalyst areas orders of magnitude larger, which are introduced further down), which also had a low relative consumption of O_2_, as shown by mass spectrometry (Figure S6.1d). Both the single particle and the large-area samples are on flat substrates, which means there is an excessive gas volume that is not in contact with catalyst material, thus in both cases only a small fraction of the total gas flow is consumed.

From analyzing the critical O_2_ exposure for 275 single particles from 4 samples, we note that, on average, polycrystals start the subsurface oxidation at a lower critical O_2_ exposure compared to single crystals (Figure 4c). This indicates that grain boundaries indeed can facilitate oxide island nucleation. The mean (and standard deviation) of the critical O_2_ exposure for subsurface oxide formation in polycrystals is (5.9 ± 3.0) × 10^8^ L, compared to (10.7 ± 4.7) × 10^8^ L in single crystals. As a reference, we can compare these numbers with the study by Lahtonen et al. where they found that at 100 °C the oxide island growth on Cu(100) started after an accumulated pure O_2_ exposure of 6 × 10^5^ L.^38^ This value is 3 orders of magnitude lower than what we observe under CO oxidation reaction conditions. However, it is a different experimental setup which might also affect the absolute exposure tolerances. Likely, there are two reasons why our particles have a higher O_2_ exposure tolerance until the onset of subsurface oxide formation that we observe under CO oxidation reaction conditions; (i) initially, CO covers the surface and either subsequently desorbs or is removed by being oxidized to CO_2_, which (ii) results in more sites on the surface that are available for oxygen adsorption, and there is a competition between oxidizing CO or the Cu surface, which means that not all adsorbed oxygen will oxidize the Cu surface.

To support this hypothesis and verify that the reaction CO + 1/2 O_2_ → CO_2_ indeed takes place at a sizable rate on our particles, we measured the CO_2_ formation rate on a large-area sample, with an estimated catalyst surface area of around 10^14^ nm^2^. This is necessary since the catalyst surface on our single particle array samples (estimated to be 5 × 10^6^ nm^2^) is too small to yield a detectable CO_2_ signal. Specifically, we made quasi-random arrays of a large number of 140 nm Cu nanoparticles by hole-mask colloidal lithography^40^ onto oxidized silicon substrates with 6.3 × 10.5 mm^2^ total area. When exposing these samples to CO oxidation reaction conditions (0–0.2% O_2_ in 0.4% CO at 250 °C) using the pocket reactor setup we have reported earlier,^7,41^ we indeed measure sizable catalytic activity (Figure S6.1). Furthermore, to exclude that the measured critical O_2_ exposure solely depends on the desorption rate of CO from the Cu particles’ surface, thereby allowing O_2_ to chemisorb and dissociate, we have investigated the critical O_2_ exposure dependence on the CO partial pressure. Specifically, we conducted corresponding experiments to Figure 4a at CO concentrations of 0.2, 0.4, and 1%, which is more than 10 times lower than the 5% CO used above. Clearly, the critical O_2_ exposure is independent of the CO concentration in this range (Figure S7.1), and we can therefore conclude that the delayed oxidation onset compared to pure O_2_ is due to the removal of adsorbed O from the Cu surface by oxidizing CO, and not due to the CO desorption rate.

A second aspect of interest, in addition to the observed earlier oxide formation onsets in polycrystals, is that in the polycrystals the subsurface oxide nucleates at 2–7 distinct sites, whereas this number is only 1–3 for the single crystals, as evident from ADF-STEM images of 61 particles acquired after 25 min into the reaction scheme (examples in Figure S8.1–8.2). We argue that this can be rationalized by the fact that we start our experimental sequence with a CO-covered Cu surface, and when introducing O_2_, CO can either desorb in its molecular form or react with oxygen and desorb as CO_2_. This is in line with the observation of immediate CO_2_ conversion upon first O_2_ exposure in our mass spectrometry experiments on large-area Cu samples (SI Section S6), which confirms that the surface is not poisoned. Hence, partly removing initial CO by conversion to CO_2_ allows more O to chemisorb, which is necessary for Cu oxide nucleation. As more O_2_ adsorbs and dissociates, there is a competition between oxygen reacting with CO forming CO_2_, or oxygen reacting with the Cu surface and thereby oxidizing it. Therefore, it is a reasonable assumption that, depending on the local distribution of surface sites for some particles (or regions on particles), this competition will lean more toward CO oxidation and on others toward Cu surface oxidation, as we discuss in more detail below on the basis of first-principles calculations.

### Energy Landscape of the CO Oxidation Reaction

**Figure 5:**
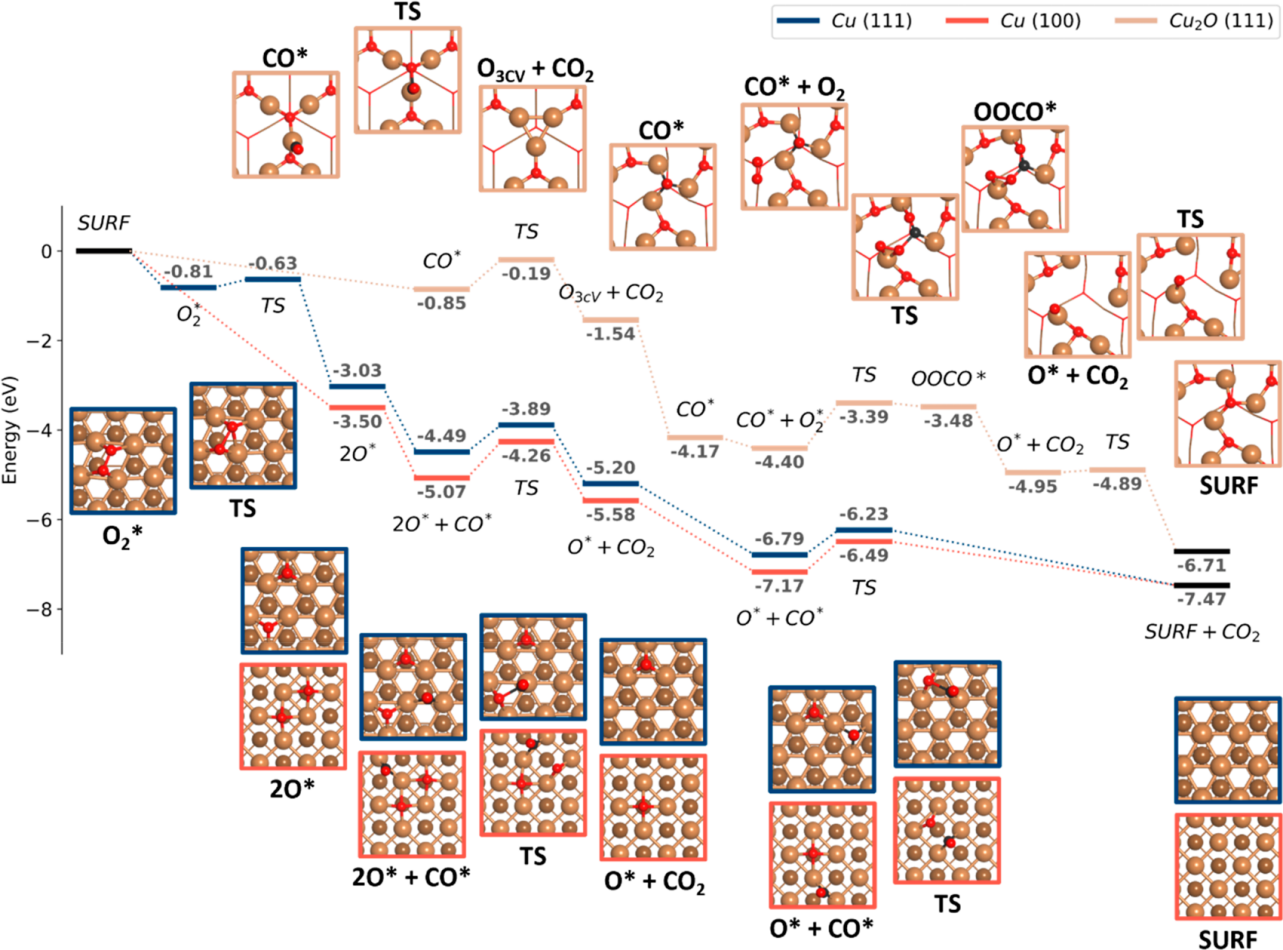
CO oxidation reaction pathways on metallic and oxidized Cu. Reaction diagrams for the CO oxidation reaction on: Cu(111) (blue), Cu(100) (red), and Cu_2_O(111) O-terminated (1 × 1) V_CuCUS_ (beige) surfaces. Top surface views are provided, with bulk atoms hidden to ease the reaction visualizations (complete slab models available in the Methods Section). In all cases, TS stands for transition state. The Langmuir–Hinshelwood mechanism was considered on the metallic surfaces and the Mars–Van Krevelen mechanism on the oxidized surface. Detailed top and side views of each reaction step can be found in Figure S10.1–10.3.

To better understand the role of different active sites in the observed Cu oxidation trends, we computed the CO oxidation reaction energy profiles for the Cu (100) and (111) facets (Figure 5). These facets were selected since they are the most abundant on the experimentally studied Cu nanoparticles. Moreover, since both facets have different surface atom coordination numbers (coordination of 8 and 9 for the (100) and (111) facets, respectively), these surfaces serve as models to understand the oxidation process in open (less coordinated) and closed (more coordinated) environments. In addition to these two metallic Cu facets, CO oxidation was also studied on the fully oxidized, O-terminated (111) (1 × 1)-V_CuCUS_ Cu_2_O facet. This surface, lacking coordinatively unsaturated Cu atoms, has been identified as the most stable surface at the experimental conditions of interest here^42−44^ (see the Methods Section for more details, and detailed top and side views in Figure S10.1–10.3). Regarding the studied CO oxidation mechanisms, for the two metallic Cu facets, we considered the Langmuir–Hinshelwood mechanism while, for the oxidized surface, we studied the Mars–Van Krevelen mechanism.

Focusing first on the metallic surfaces, we found that CO chemisorbs with adsorption energies of −1.77 and −1.61 eV on the (100) and (111) facets, respectively. Furthermore, O_2_ molecules were found to dissociate without an activation barrier on the open (100) surface, whereas, on the close-packed (111) surface, a low activation barrier of 0.18 eV was found. The dissociated O atoms chemisorb with average adsorption energies per oxygen atom of −1.75 and −1.51 eV on the (100) and (111) surfaces, respectively. The computed trends on O_2_ adsorption and dissociation agree with previous calculations on low-index Cu surfaces.^45^ Regarding the two studied CO oxidation steps (Figure 5), we found lower activation barriers on the close-packed (111) surface (0.60 and 0.56 eV), compared to the (100) surface (0.81 and 0.68 eV). Hence, although both the adsorption of reactants and the dissociation of molecular oxygen are preferred on the low-coordinated surface, the kinetic barriers for CO oxidation are lower on the high-coordinated one, in agreement with previously identified Brønsted–Evans–Polanyi (BEP) relations for Cu systems.^46^

Two conclusions can be made based on the identified trends in adsorption, O_2_ dissociation, and CO oxidation that can help explain the critical O_2_ exposure distributions in Figure 4c, in which polycrystals were found to have a lower O_2_ exposure tolerance before reaching *t*_20_. First, the low-coordinated sites found at the polycrystals’ grain boundaries facilitate the dissociation of O_2_ molecules which, in addition to the hindered CO oxidation at the low-coordinated sites, means that more O atoms will be available for Cu oxidation. On the contrary, for single crystals rich in high-coordinated sites, the reverse kinetic trends mean that the balance is pushed toward CO oxidation, delaying the Cu oxide nucleation process. In this discussion, it is worth mentioning that the surfaces are precovered with CO before O_2_ starts to flow, however, despite the strong CO binding energies computed, CO does not appear to poison the catalyst. We observe CO_2_ production immediately when the O_2_ flow is introduced (Figure S6.1).

Finally, focusing on the oxidized surface, we obtained higher activation energies for the CO oxidation elementary steps compared to the metallic surfaces. Hence, CO oxidation will be kinetically preferred on the metallic Cu surfaces. This could also help explain the decrease in CO_2_ production toward a lower, steady state as the O_2_ concentration increases (Figure S6.1). In other words, after the initial rapid production of CO_2_, surface oxidation begins and reduces the CO_2_ production gradually until it reaches a lower bound, corresponding to the oxidized surface’s production (in agreement with previous observations on similar particles^6^). As a final note, the total reaction energies (Δ*E*_reaction_ = 2*E*_CO_2__ – 2*E*_CO_ – *E*_O_2__) in the oxidized and metallic systems have a difference of about 10%. This discrepancy is caused by the implementation of the Hubbard *U* correction^47,48^ on O’s p orbitals for accurate simulations of the oxide surface structural and electronic properties and is discussed further in the SI Section S10.

To further elucidate the role of grain boundaries and defect sites in the oxidation of Cu, we also studied the surface diffusion energies of single O atoms. Previous computational work has shown a decrease in O-diffusion barriers as the number of Cu–O bonds along a diffusion trajectory increases.^49^ This results in diffusion barriers that increase in the order of the (110), (111), and (100) facets of Cu, as well as a general trend for upward diffusion paths toward the terraces of step-edge sites.^49^ In this study, we compared the diffusion energies for the (100) and (111) Cu facets with those on grain boundary sites.

Considering there’s a plethora of grain boundary structures to choose from, we focused on two slab models with opposing atomic fit levels, i.e., with differing number of coincidental sites and general overlap between grains. First, we studied the highly symmetric Cu coherent twin grain boundary (CTB), comprised of two perfectly overlapping (110) grains (more information in the Methods Section). The surface atoms at the CTB and outside of it have the same coordination numbers (provided in Figure S10.5), which leads us to expect a similar chemical behavior inside and outside of the boundary. Besides the symmetric CTB, we also studied O diffusion around a Cu (111) step-edge surface with a {111} microfacet (see Methods Section). The step-edge model was used as a surrogate of a low-coincident boundary, with large differences in its surface atoms’ coordination numbers (Figure S10.5). As an initial result, for both the CTB and the step-edge slabs, O_2_ molecules were found to dissociate without an activation barrier, agreeing with the previously found barrierless dissociation on low-coordination sites.

**Figure 6:**
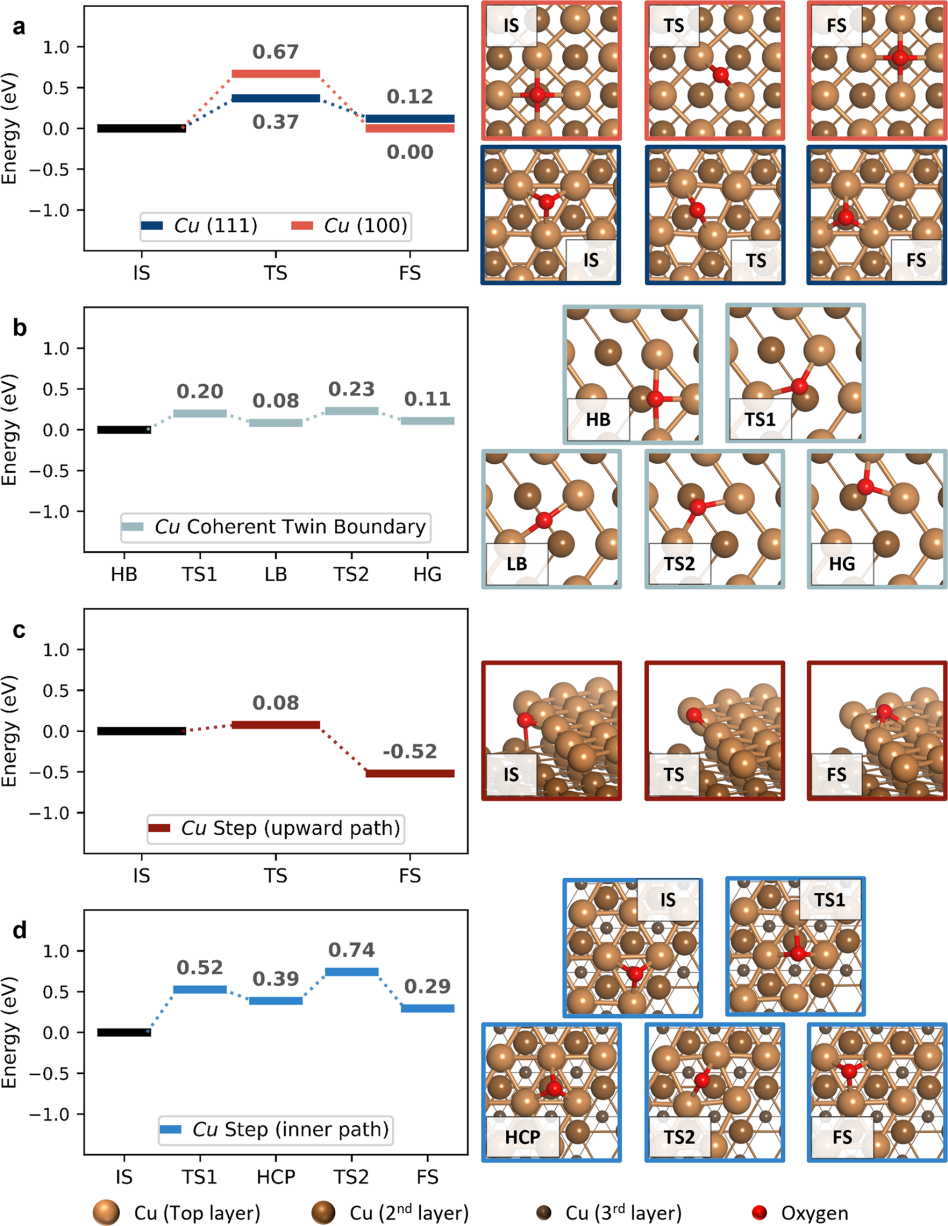
The kinetics of O atom diffusion. Oxygen diffusion energies on Cu (a) (100) and (111) facets, (b) coherent twin boundary, (c) (111) step-edge surface, going from the lower to the upper terrace (upward path), and (d) top terrace of (111) step-edge, diffusing first inward to an adjacent hcp site (step 1), and then to an adjacent fcc site (step 2). More information about the chosen slab models can be found in the Methods Section. In all cases, IS, TS, and FS stand for initial, transition, and final state, respectively. As for the stable intermediates in (b) LB stands for long-bridge position, found between two hollow sites on the coherent twin boundary slab, and in (d) HCP is the hollow hcp site on the upper terrace of the step-edge slab. In (b) HB and HG stand for hollow sites on the boundary and on the grain outside of it, respectively, whilst LB stands for the long-bridge position found in between them.

First, analyzing the diffusion barriers on the (100) and (111) facets (Figure 6a), we obtained lower diffusion barriers on the (111) facet compared to the (100) facet, in agreement with previous literature results.^49^ Furthermore, no net energy change was noted for the (100) facet since diffusion was studied between two equal hollow sites. For the (111) facet, a slight net energy increase was observed for diffusion from a hollow face-centered cubic (fcc) site to a less-preferred hexagonal close-packed (hcp) site. Focusing next on the CTB (Figure 6b), we observed lower diffusion barriers compared to the (100) and (111) facets. This is natural considering that the CTB is comprised of two perfectly overlapping (110) facets, which have lower diffusion barriers compared to the (100) and (111) facets.^49^ Moreover, we found a small net energy increase (0.11 eV) for O diffusing out from the CTB. Analogously, we justify this energy difference by the number of Cu atoms the O atom binds to, which is three in the hollow boundary site on top of the CTB and two outside the boundary. This small change influences the reaction barriers, and thus the preferred diffusion path of O atoms is toward the boundary.

Regarding the (111) step-edge model (Figure 6c,d), we considered two O diffusion paths: an upward diffusion path (Figure 6c), in which O diffuses from the lower to the upper terrace, and a diffusion path on the upper terrace, in which O diffuses through the hollow sites of the upper terrace (Figure 6d). The upper terrace path includes two steps: O diffusing away from the edge of the terrace to a neighboring hcp site, and from there to a neighboring fcc site.

We note that the diffusion barriers are considerably lower in the direction of the low-coordinated step-edge atoms, compared to diffusing away from these toward more coordinated atoms. This can be seen in both the upward diffusion path (0.08 eV diffusing upward, toward the step-edge atoms vs 0.60 eV away from them), as well as in the first upper diffusion path (0.13 eV toward the edge vs 0.52 eV away from the edge). In both considered diffusion paths on the step-edge slab, O binds to a larger number of low-coordinated Cu atoms at the edge of the step. Moreover, it is worth noting that, for the second considered diffusion step in the upper (111) terrace, the diffusion barriers and energies resemble those of the (111) surface.

To summarize the oxygen diffusion computational results, we observe that O atom diffusion toward grain boundary sites is favored. Hence, grain boundaries, especially those rich in defects and low-coordinated sites, will tend to trap O atoms. This effect, together with the barrierless dissociation of O_2_ molecules on low-coordinated sites, explains why surface oxidation is enhanced along grain boundaries (Figure 3, Figure S4.1), and why more oxide nucleation positions are found on polycrystals (Figure S8.1–8.2). Hence, subsurface oxidation can be understood by the interplay of multiple factors: the readiness to dissociate O_2_ molecules, the consumption of O atoms by the competing CO oxidation reaction, and the diffusion of O atoms toward nucleated oxide islands.

### Dependence of the Critical O_2_ Exposure Limits on the Grain Boundary Density

**Figure 7:**
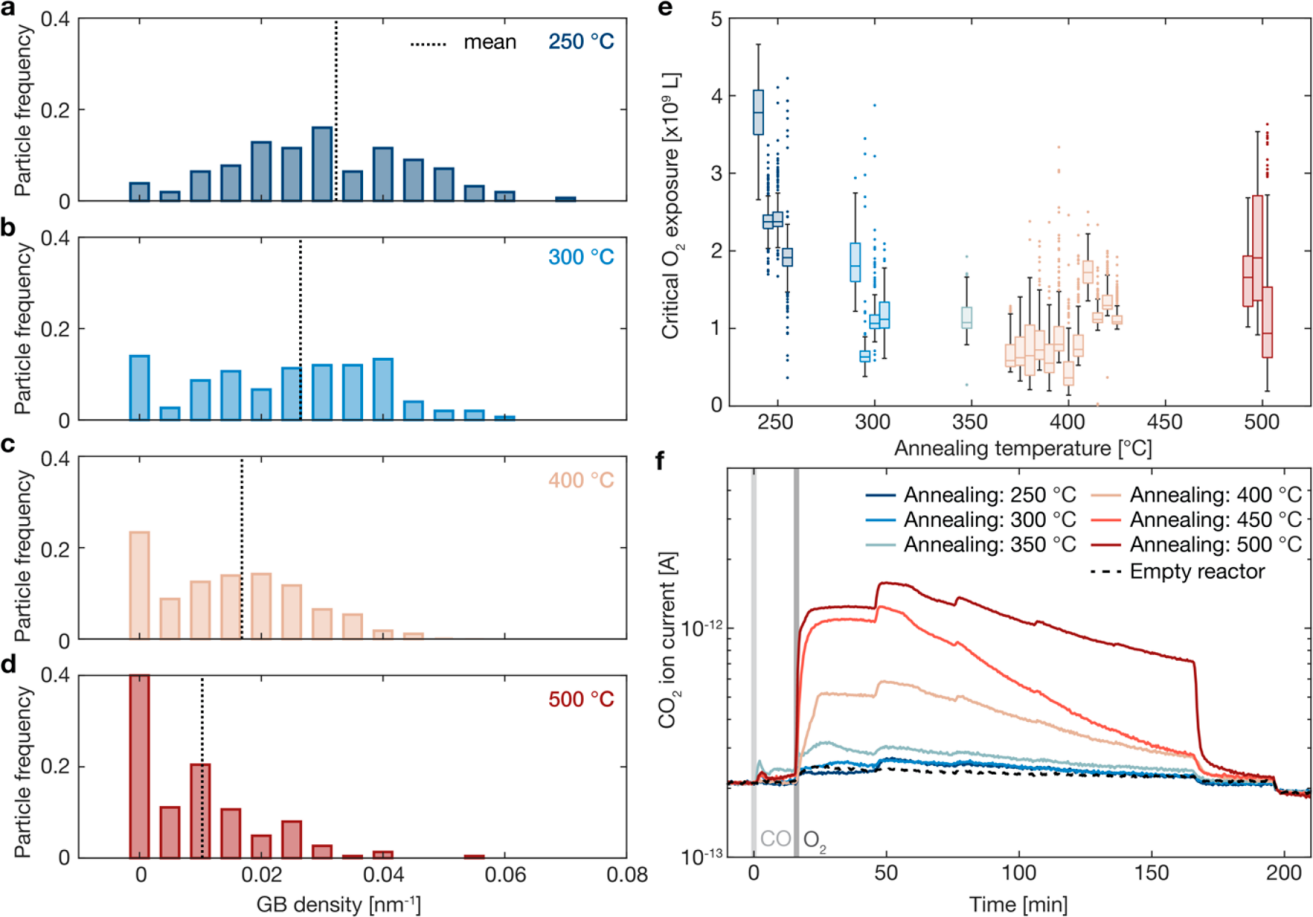
Dependence of the critical O_2_ exposure on the annealing temperature. The distribution of grain boundary densities found in particles thermally annealed at (a) 250 °C, (b) 300 °C, (c) 400 °C (same data as in Figure 1d) and (d) 500 °C, with the dashed lines indicating the mean for each temperature. The distributions are normalized by the number of particles in each set: 156, 150, 878, and 225 particles, respectively. (e) The mean critical O_2_ exposure from each separate CO oxidation experiment on single particle samples thermally annealed at either 250, 300, 350, 400, or 500 °C. The line in the box is the mean and the box contains 50% of the particles in each sample. Some of the data points are shifted slightly left or right so that all data are visible. (f) The CO_2_ ion current measured from large-area samples while stepping up the O_2_ concentration from 0% to 0.2% in steps of 0.04 percentage points in a background of 0.4% CO and at 250 °C. The samples are pretreated at the temperatures indicated: 250, 300, 350, 400, 450, and 500 °C for 2 h in 2% H_2_ in Ar. A reference measurement with an empty reactor was done under the same conditions (dashed line). The start of the CO (0 min) and O_2_ (15 min) flows are marked by the gray lines.

To further study the effect of the grain boundary densities, we have extended our study of the critical O_2_ exposure before the onset of Cu oxidation to a set of 24 experiments on separate samples with varying pretreatment temperatures. We used the same type of particle arrays as shown in Figure 1a and S1.1. The samples were thermally annealed at either 250, 300, 350, 400, or 500 °C to control the distribution of the particle grain boundary densities in the samples (Figure 7a–d). We note that as the annealing temperature increases, the distributions become increasingly skewed toward the low grain boundary densities, with a noticeable increase in the number of single crystals. Hence, as the annealing temperature increases, the surface coordination of the samples increases. The mean and standard deviation of the particles’ grain boundary densities in samples annealed at 250 °C was 0.032 ± 0.015 nm^–1^, at 300 °C it was 0.026 ± 0.016 nm^–1^, at 400 °C it was 0.017 ± 0.013 nm^–1^, and at 500 °C it was 0.010 ± 0.011 nm^–1^. These numbers were calculated from sets of 156, 150, 878, and 225 particles, respectively.

When studying the critical O_2_ exposures in these samples, calculated as the total O_2_ exposure at *t*_20_, we note two trends. In general, samples annealed between 250 and 400 °C exhibit decreasing critical O_2_ exposure limits with increasing annealing temperatures (Figure 7e). Strikingly, the general trend flips at 400 °C and, at the highest annealing temperature of 500 °C, the critical O_2_ exposure limit is instead pushed to higher exposures. To understand this nonlinear behavior, we again turn to pocket reactor experiments and our large-area samples described above, which we now pretreated at the same range of annealing temperatures prior to the CO oxidation conditions at 250 °C (Figure 7f). The most noticeable effect is the increasing CO_2_ production on the samples annealed at 400–500 °C, compared to samples pretreated at lower temperatures. This effect is in line with our DFT-calculated activation energies (cf. Figure 5), which showed a higher activity for CO oxidation on high-coordinated sites that are more abundant on samples with fewer grain boundaries. The second effect we notice in the samples annealed at lower temperatures that have more grain boundaries (most distinctly seen in the sample annealed at 250 °C, dark blue line in Figure 7f) is that the onset of CO_2_ conversion is delayed compared to samples annealed at 400–500 °C. At the start of the O_2_ flow, the CO_2_ production rate from the high-temperature pretreated samples is immediately increasing and reaching a maximum after between 5 and 10 min for the 400, 450, and 500 °C annealed samples. However, for the 250 °C treated sample, the CO_2_ production is not elevated above the baseline of the empty reactor until increasing the O_2_ concentration to 0.08%, and a maximum is reached after about 35 min. Based on our previous discussion, we attribute this delay to slower CO desorption in the samples annealed at low temperatures, in which low-coordination sites at the grain boundaries, as well as other defects that bind strongly to CO,^50^ are more abundant. This is in line with CO temperature-programmed desorption from Cu(410) surfaces, where CO desorption from the edge site was found to a have higher activation energy than from facet sites.^51^ As mentioned before, CO desorption is required to allow O_2_ to dissociate on the surface and sequentially oxidize CO.

In summary, when increasing the pretreatment temperature, the density of high-coordination sites in each particle increases, which eases the desorption of CO, as reflected in both a rapid increase in the production of CO_2_ and a higher maximum CO_2_ production rate. Additionally, in the samples pretreated at high temperatures, the CO oxidation reaction rate is high, which reduces the number of available O atoms for oxidizing the Cu surface. On the contrary, in samples pretreated at low temperatures, the density of low-coordination sites is high. Therefore, in this sample population pretreated at low temperatures, there are more sites that adsorb CO strongly, which delays the Cu surface oxidation onset because desorbing CO takes longer time. Hence, in the samples with an abundance of low-coordination sites, the strong CO adsorption is reflected in the delay until reaching the maximum CO production, which translates into the delay of the subsequent Cu surface oxidation onset.

## Conclusions

**Scheme 1:**
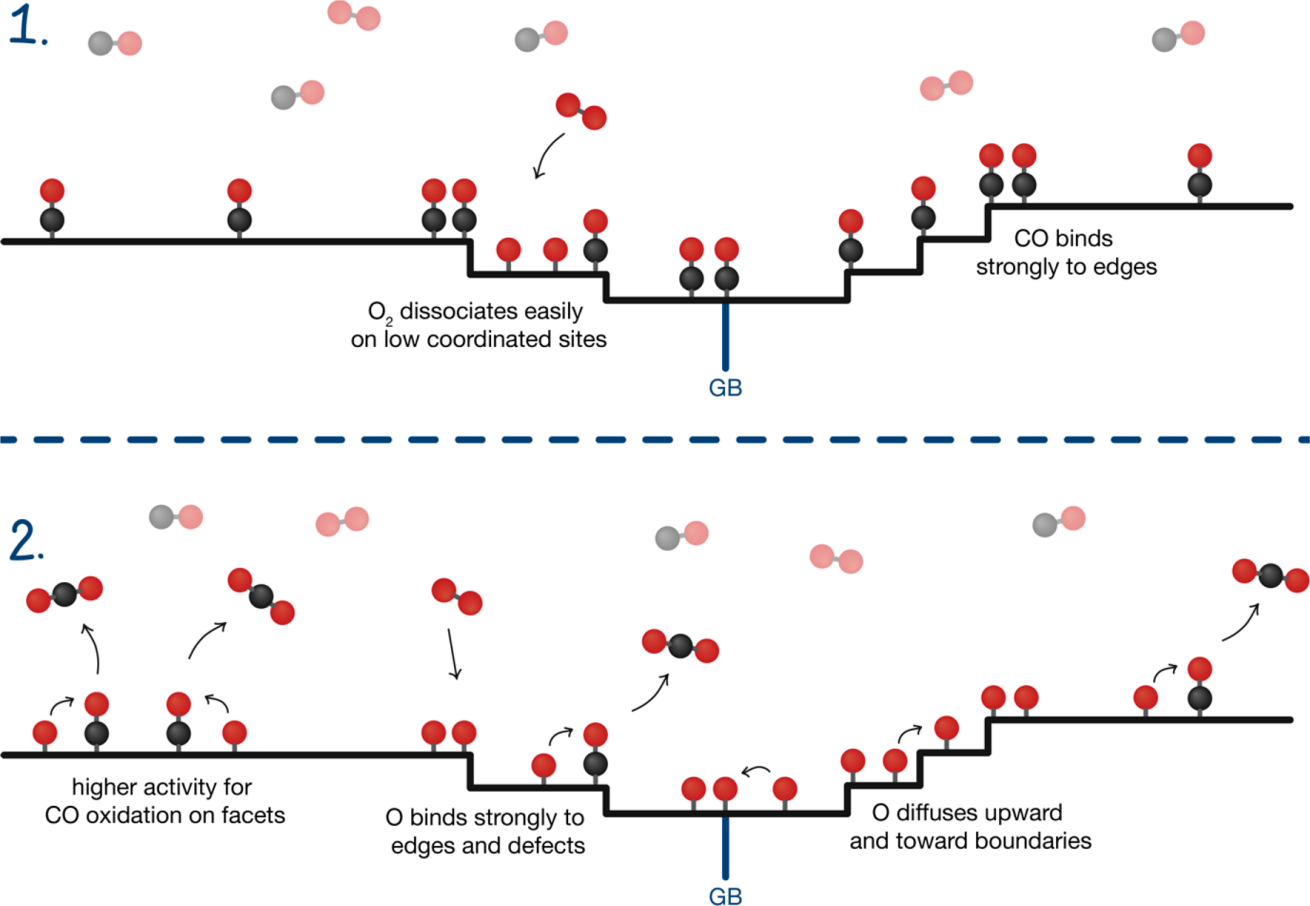
The Main Contributing Aspects to Site Specific Cu Nanoparticle Surface Oxidation during the CO Oxidation Reaction (1) We are starting out with a CO covered surface. CO is removed by oxidizing to CO_2_ and desorbing, or desorbing as CO. CO binds more strongly to low coordinated sites, which can initially delay surface oxidation of these sites. (2) The CO oxidation reaction is enhanced on closed surfaces, like Cu(111). Furthermore, O_2_ dissociates more easily on low coordinated sites, as well as diffuses preferentially upwards and towards grain boundaries, which provide O atom sinks at steps and boundaries.

Using TEM image analysis, we have characterized the grain boundary density of 1418 Cu nanoparticles out of a total of 5040 particles studied with *in situ* plasmonic nanoimaging under CO oxidation reaction conditions. By analyzing the decrease in the relative light scattering intensity for the TEM-imaged particles, we have identified a clear dependence of the onset of Cu particle oxidation along the time axis on the grain boundary density under CO oxidation reaction conditions. Furthermore, *ex situ* ADF-STEM images revealed that oxide nucleation occurs at a limited number of sites on the particle surfaces, which leads to a nonuniform oxide growth that suppresses Kirkendall void formation. These oxide nucleation sites are preferentially located in the vicinity of grain boundaries. Moreover, the grain boundaries enhance the oxide growth rates, leading to apexed oxide growth fronts in polycrystalline particles, compared to straight oxide growth fronts observed mainly in single crystals. We validated the oxide nucleation trends by DFT (summarized in Scheme 1), identifying that (i) O_2_ dissociation is favored on low-coordinated sites, which also bind stronger to O atoms and therefore tend to trap O, (ii) O diffusion is favored toward steps and along grain boundaries, and (iii) high-coordinated metallic Cu sites enhance the CO oxidation reaction.

We further analyzed the effect of the annealing temperature, and thereby the effect of grain boundaries since they are more abundant in the low-temperature pretreated samples, on the O_2_ exposure necessary to initiate the oxidation of the Cu particle surface. We found a nonlinear trend where both the samples annealed at low (250 °C), and high (500 °C) temperatures could tolerate higher O_2_ exposures before oxidizing, compared to those annealed at moderate temperatures (350–400 °C). This can be understood from the first-principles insights summarized above, and visually depicted in Scheme 1. Specifically, at elevated annealing temperatures, high-coordinated sites are more abundant, which have enhanced CO oxidation rates, thus consuming O atoms and delaying the surface oxidation. On the other hand, low-temperature pretreated samples (rich in low-coordinated sites) bind CO strongly, which, for precovered surfaces, also delays the oxidation onset, despite the enhanced O_2_ dissociation and O diffusion rates. On the contrary, for samples annealed at moderate temperatures, none of the two effects that delay oxidation is strong, and instead the O_2_ dissociation and O trapping on low-coordinated sites dominate, which leads to low O_2_ tolerance.

In summary, our findings emphasize the importance of site engineering in catalysis to make more active and lasting catalyst particles. Specifically, we have shown that some sites can lead to high activity and others to catalyst deactivation over time. In our case of CO oxidation over Cu catalysts, nanoparticles with high-coordination sites and few grain boundaries (and other defects) will make the catalysts last longer before surface oxidation and thus deactivation. Furthermore, we highlight that it was crucial for our study to characterize the grain morphology of a large number of single particles. This allowed us to detect trends among the heterogeneity in the oxidation rates we observed for the individual Cu nanoparticles, which stem from the structural complexity of the particles’ surfaces. This complex system emphasizes the importance of a statistically relevant sample size to link single particle results to particle ensemble behaviors typically observed in industrial catalysts. In this way, our study further strengthens the position of correlative TEM and plasmonic nanoimaging as a concept to bridge the “materials gap” in catalysis research at the single nanoparticle level^52^ when also combined with DFT calculations. Looking forward, we also predict its application in other areas, such as electrochemistry,^19,20^ battery research,^53^ sensing applications,^54^ nanosafety^55,56^ and nanomedicine,^57^ where structure–function correlations are of central importance.

## Methods

### *In Situ* Dark-Field Plasmonic Nanoimaging

The *in situ* plasmonic nanoimaging experiments were conducted in a Linkam reaction chamber (THMS600) with optical access, mounted on an upright Nikon Eclipse LV150 N microscope and connected to mass flow controllers (Bronkhorst, low-ΔP-flow and EL-flow) to regulate the supplied gas mixture at atmospheric pressure using 15% (±2 rel.%) O_2_ in Ar (6.0 purity) or 100% H_2_ diluted to 2% total concentration in Ar carrier gas (6.0 purity). To enable plasmonic nanoimaging in dark-field scattering configuration, the microscope is equipped with a dark-field objective (Nikon TU plan ELWD 50×, NA = 0.60, WD = 11 mm) and light was collected by an Andor Newton 920 CCD camera (256 × 1024 pixels). The scattering image, *F*_raw_, from the particles illuminated by a 50 W halogen light source, was collected every 5 or 10 s (0.25 s exposure with 15 or 30 accumulations). The CCD dark current, *F*_dark_, was collect as an image without illuminating the CCD and subtracted from the raw image frames to yield the scattering intensity frames as *F*_scat_ = *F*_raw_ – *F*_dark_. A particle finding algorithm based on a wavelet filter was used in the first frame and every sequential frame was stabilized with respect to the first frame. In each frame, *F*_scat_, the normalized scattering intensity per pixel at each particle, was collected from a box of 7 × 7 pixels and normalized as *Ĩ*_*p*_ = *I*_*p*_/49, and the background scattering was collected in a frame of 9 × 9 pixels outside the particle box, *Ĩ*_*b*_ = *I*_*b*_/32, thus *Ĩ*_Cu_ = *Ĩ*_*p*_ – *Ĩ*_*b*_. On the sample chip, there was an array of 25 or 30 Au nanoparticles (depending on the sample design), which served as an optical reference to account for illumination intensity fluctuations in the images. Specifically, the scattering intensity of the Au particles, *Ĩ*_Au_, was collected equivalently to the Cu particles with a corresponding background intensity. Finally, the scattering intensity from Cu nanoparticle *j* during oxidation was calculated as *Ĩ*_*j*_ = *Ĩ*_Cu,*j*_/mean(*Ĩ*_Au_).

### Sample Nanofabrication

TEM compatible substrates comprising of a 25 nm thin SiN_*x*_ film grown on a Si wafer, in which a 120 × 120 μm opening was etched, were fabricated in-house following the recipe developed by Grant et al.^22^ By means of electron beam lithography, arrays of 5 × 5 nanoparticles were fabricated onto these silicon nitride thin film membranes according to the following fabrication procedure: (1) A thin film of copolymer MMA(8.5) MMA (MicroChem Corporation, 10 wt % diluted in anisole) was spin coated at 6000 rpm for 60 s and followed by baking on a hot plate at 180 °C for 5 min. This was followed by spin coating PMMA A2 at 3000 rpm for 60 s and baking at 180 °C for 5 min. (2) The resist was patterned by electron-beam exposure in a JEOL JBX 9300FS (2 nA with a shot pitch of 2 nm, 2000 mC/cm^2^ exposure dose). (3) The pattern was developed in methyl isobutyl ketone (MIBK):isopropanol (1:3) for 120 s, followed by drying under N_2_-stream. (4) Either a 20 nm Au or a 40 nm Cu thin film, depending on if fabricating the Au reference particles or the Cu nanoparticles, was deposited by electron beam evaporation in a Lesker PVD 225 at a rate of 1–2 Å/s and lift-off was done in acetone for approximately 12 h. (5) Finally, to achieve the desired grain morphologies, the sample was annealed in 2% H_2_ in Ar at 400 °C for 1–4 h.

### Transmission Electron Microscopy Image Acquisition and Analysis

Imaging of Cu nanoparticles before (annealed state) reaction conditions was conducted in a FEI Tecnai T20 with LaB6 filament, operated at 200 kV. The sample was taken directly from the reaction chamber after thermal annealing (400 °C in 2% H_2_/98% Ar) to the microscope to minimize hydrocarbon contamination, which later can be deposited and polymerized by the electron beam. The imaging was done in bright field-mode at a magnification of 71 kX using an objective aperture to reduce diffraction ghost images. Imaging after reaction experiments was done by means of annular dark-field scanning transmission electron microscopy (ADF-STEM) in an aberration corrected FEI Titan 80–300 with a field emission gun operated at 300 kV. The ADF-STEM imaging was done at a camera length of 195 mm.

From the bright-field TEM micrographs of thermally annealed copper nanoparticles the grain boundary length was measured in MATLAB (version 2020b) using the image processing toolbox (function *drawassisted*). The relative error of the grain boundary length measured from TEM images, *L*_TEM__,_ compared to the grain boundary length obtained from transmission Kikuchi diffraction (TKD), *L*_TKD_, was calculated as RE = (*L*_TEM_ – *L*_TKD_)/*L*_*TKD*_. The particle area before oxidation was measured as follows. The images were morphologically filtered (function *imopen*, structure element: disk, radius 5 pixels) to reduce noise close to the particle edge before the outer perimeter of the particle was detected (using *bwboundaries*). A circle was then fitted to the detected perimeter to calculate the particle radius *R*. The remaining metallic Cu area after 55 min at reaction conditions was measured in ImageJ.

### Pocket-Reactor Quadrupole Mass Spectrometry

To quantify the reaction products on the large-area samples we used a plug flow-type reactor (X1, Insplorion AB, Göteborg, Sweden) connected to a quadrupole mass spectrometer (QMS, GSD 320, Pfeiffer). The so-called large-area sample constitutes a dense particle array on a 6.3 × 10.5 mm^2^ Si substrate, which was fabricated following the hole-mask colloidal lithography protocol.^40^ The sample was placed inside a custom-made glass pocket, as reported by Bu et al.^7^ and the setup used in this work was previously reported by Tiburski et al.^41^ The temperature was read out by a K-type thermocouple at the sample position connected to a PID temperature controller (Eurotherm 3508), and the reactor was heated by a resistive heating coil. The gas flow into the reactor was controlled by mass flow controllers (low−Δ*P*–flow, Bronkhorst). The gases used were O_2_ (99.9992% purity), 4% H_2_ (±2%) in Ar, and 10% CO (±2%) in Ar, with Ar (6.0 purity) as the carrier gas.

### Computational Methods

Spin-Polarized Density Functional Theory (DFT) calculations were performed on the CP2K^58^ software package using the Quickstep approach.^59^ Atomic visualizations were generated with VESTA.^60^ The Perdew–Burke–Ernzerhof (PBE) exchange-correlation functional^61^ was used in our DFT calculations. Dispersion effects were accounted for using Grimme’s D3 dispersion correction method.^62,63^ Molecularly optimized (MOLOPT) DZVP basis sets^64^ were used alongside auxiliary planewave basis sets, defined with a kinetic energy cutoff of 600 Ry. Core–valence interactions were considered using the pseudopotentials of Goedecker, Teter and Hutter (GTH).^65,66^ For Cu_2_O, a Hubbard *U* correction was applied using Dudarev’s formulation^48^ with a *U*–*J* (*U*_eff_) value of 3.0 eV on Cu’s d orbitals and of 2.0 eV on O’s p orbitals. These *U* values were chosen to better replicate the cell parameters of Cu_2_O. For all systems, 3 × 3 × 3 supercells, based on the respective conventional cells, were chosen. The optimized lattice structures agree with reported experimental values within 5%. Bulk optimization details are summarized in Table S10.2.

Concerning the geometric optimizations, 15 Å of vacuum and dipole corrections^67^ were used, while the force cutoff was set to 4.5 × 10^–4^ Ha Bohr^–1^. For Cu, slab models of the most stable (111) in addition to the (100) facets were used, respectively keeping the bottom 3 and 2 layers frozen at their corresponding bulk positions. Besides these two facets, a stepped Cu (111) surface was simulated, which was built by removing one row of the Cu (111) slab model to expose the {111} microfacet, keeping the number of frozen layers intact. To further gain insights about the role of grain boundaries, the surface of Cu’s coherent twin boundary, experimentally determined to be one of the most abundant grain boundaries in Cu’s surfaces,^68−70^ was also studied. This boundary, defined under the coincidence site lattice (CSL) theory, is specified by a multiplicity index, Σ = 3, the {111} grain boundary plane and the ⟨111⟩ rotation axis. The symmetric grain boundary slab was built using the Aimsgb python package,^71^ keeping the bottom four layers fixed at their bulk positions. With regards to Cu_2_O, the nonstoichiometric (111) O-terminated (1 × 1) V_CuCUS_ facet, generated by removing the coordinatively unsaturated surface copper atoms, was employed as it is reported to be the most stable facet.^42−44^ In this case, the bottom 3 trilayers (copper layer enclosed by two oxygen layers) were kept frozen at their corresponding bulk positions. Representations of the used slab models are shown in Figure S10.7.

Adsorption energies (*E*_ADS_) were calculated by eq 1, where *E*_S_ and *E*_M+S_ are the energies of the slab with and without adsorbates, respectively, while *E*_M_ corresponds to the energy of an isolated adsorbate molecule.
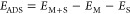
1Finally, the dimer method was used to optimize transition state geometries,^72^ which were further verified with vibrational frequency calculations (single imaginary frequency along the reaction coordinate).

## Supporting Information Available

Sample design and Au optical reference, dependence of scattering intensity on oxidation, plasmonic nanoimaging raw data and *t*_20_, enhanced oxidation along grain boundaries, STEM oxide characterization, mass spectrometry of the CO oxidation reaction over large-area sample, varying the CO background concentration, oxide nucleation positions and delayed Kirkendall void formation, control experiment without CO, density functional theory calculations (PDF)
