# Supplementary material for: The Role of Grain Boundary Sites for the Oxidation of Copper Catalysts during the CO Oxidation Reaction
Source: ACS Nano. 2023 Oct 5;17(20):20284–98. doi: 10.1021/acsnano.3c06282 (PMC10604102; doi:10.1021/acsnano.3c06282)
Supplement: Supplementary file 1 — nn3c06282_si_001.pdf [file nn3c06282_si_001.pdf]

Supporting Information

for

The Role of Grain Boundary Sites for the Oxidation of Copper Catalysts during the CO Oxidation Reaction

Sara Nilsson[1,§], John N. El Berch[2], David Albinsson[1], Joachim Fritzsche[1], Giannis Mpourmpakis[2,†] and Christoph Langhammer[1,]*

[1]Department of Physics, Chalmers University of Technology, 412 96 Göteborg, Sweden

[2]Department of Chemical and Petroleum Engineering, University of Pittsburgh, Pittsburgh, PA, 15261, USA

[§]nisara@chalmers.se

[†]gmpourmp@pitt.edu

[*]clangham@chalmers.se

CONTENT

S1: Sample design and Au optical reference

S2: Dependence of scattering intensity on oxidation

S3: Plasmonic nanoimaging raw data and t_{20}

S4: Enhanced oxidation along grain boundaries

S5: STEM oxide characterization

S6: Mass spectrometry of the CO oxidation reaction over large-area sample

S7: Varying the CO background concentration

S8: Oxide nucleation positions and delayed Kirkendall void formation

S9: Control experiment without CO

S10: Density functional theory calculations

S1: Sample design and Au optical reference

We use samples with Au nanoparticles as optical reference because they do not oxidize and thus scatter at a constant intensity throughout the experiment. Hence, by collecting the scattering intensity from the Au nanoparticles displayed in **Fig. S1.1** and **Fig. 1** in the main text, we can deconvolute the effect of the overall decreasing background scattering intensity due to oxidizing Cu from each individual particle's scattering intensity by dividing with the average Au particle scattering intensity (**Fig. S1.2**). In this way, we can also deconvolute any potential drift in the light source. This is described in detail in the **Methods Section**.

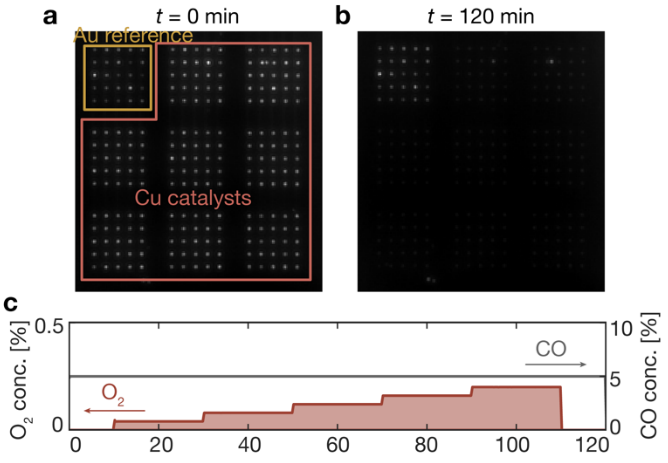

Figure S1.1: *Dark-field light scattering microscope images of the second sample design, used for the experiment presented in **Fig. 2** in the main text, taken at the start of the experiment (a) and at the end after the reaction when all Cu particles are oxidized and therefore appear dark (b). (c) CO oxidation reaction conditions were established by increasing the O_2 concentration in the feed from 0 % to 0.2 % in steps of 0.04 %, in a constant background of 5 % CO in Ar carrier gas at 250 °C and atmospheric pressure. The sample design presented in **Fig. 1** in the main text is a development of the design in this figure and has, as the main difference, Au particles both at the top and bottom of Cu nanoparticle arrays from which an average background scattering is calculated that accounts for background scattering intensity changes across the sample.*

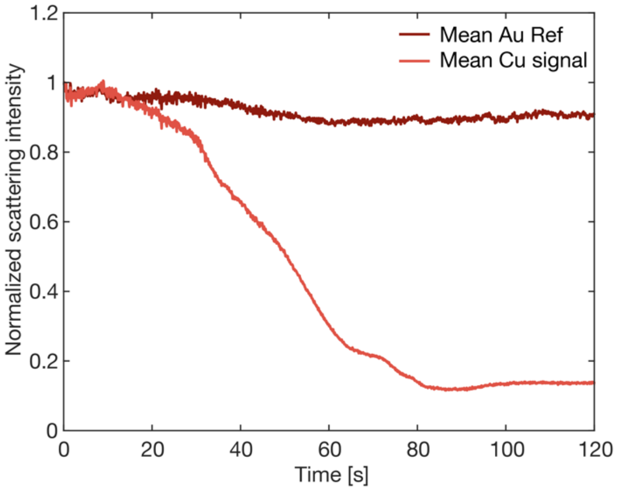

Figure S1.2: *Mean normalized scattering intensity time trace obtained by averaging the signal from 200 single Cu particles (bright red) plotted together with the mean normalized scattering intensity of the 25 Au reference particles (dark red) on the same sample (cf.* ***Fig. S1.1****). Note that also the intensity of the Au particles decrease slight over time when the Cu particles oxidize. This is not the consequence of Au oxidation but due to decreasing overall background scattering intensity on the sample when the Cu particles start to oxidize and accordingly scatter significantly less light.*

S2: Dependence of scattering intensity on Cu oxidation level

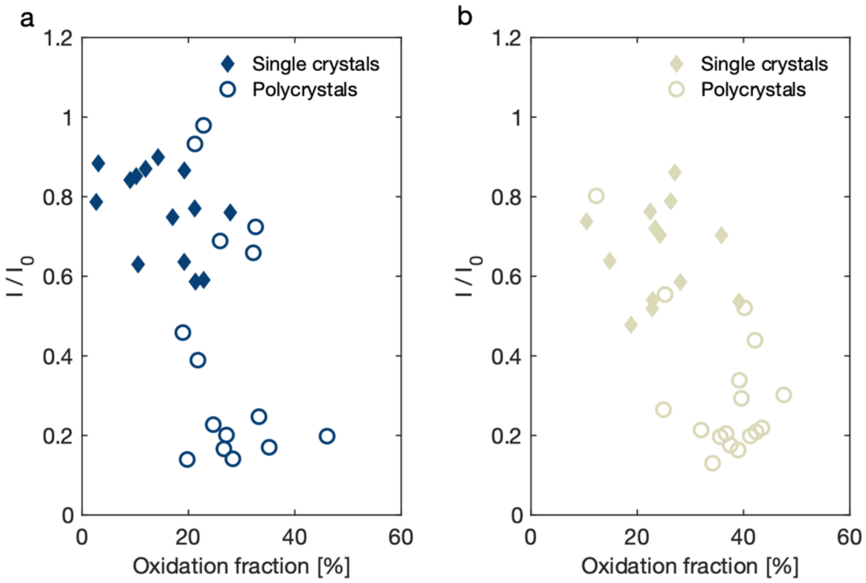

Figure S2.1: *The normalized light scattering intensity vs. the oxidation fraction obtained from two samples: (a) annealed 1 h at 400 °C and same particles as* ***Fig. S8.3-8.4****, and (b) annealed 2 h at 400 °C. The oxidation fraction is extracted from STEM images taken ex situ after exposure of the sample to CO oxidation reaction conditions. There is a roughly linear relation between the intensity decrease and the oxidation fraction, with Pearson correlation coefficients (a) -0.58 and (b) -0.67. Note that on average the single crystals (filled diamonds) are less oxidized compared to the polycrystals (empty circles) when terminating the reaction conditions. The spread in the data can have various reasons, among which the difference in the shape of the growing oxide will result in different optical response, because the shape of the remaining metal volume is directly reflected in the LSPR, and thus in the scattering intensity*[1]*. When around 40 % of the Cu particle has oxidized, the scattering from the remaining metal volume is becoming very weak and it is no longer decreasing linearly with the oxide growth.*

S3: Plasmonic nanoimaging raw data and t_{20}

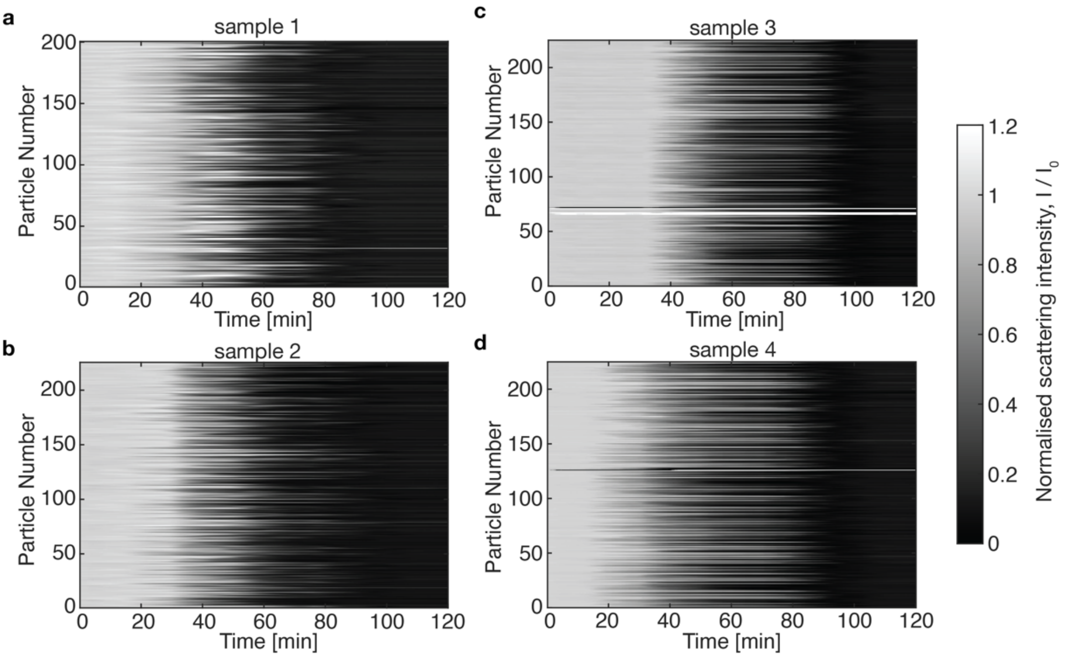

Figure S3.1: Normalized scattering *intensity maps of the four samples from which the critical O_2 exposures were extracted and presented in **Fig. 4** in the main text. The intensity map of sample 1 (a) is the same figure as in **Fig. 1e**. The particles that appear bright along the entire reaction in (c, d) are located close to a dust particle. Hence, it is the scattering from the dust that is collected and these signals are not included in **Fig. 4**. On sample 1, 2 and 4; 75 particles were imaged and classified as single or polycrystals, and included in **Fig. 4** in the main text. On sample 3; 50 particles were imaged and included in **Fig. 4**.*

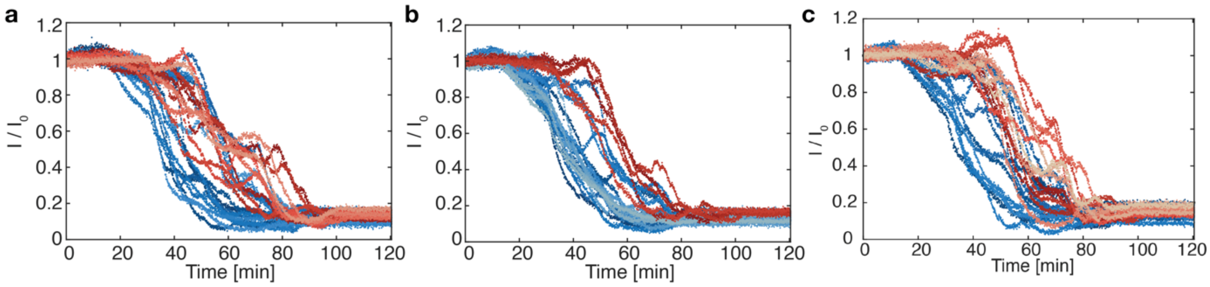

Figure S3.2: *Normalized scattering intensity time traces of 75 particles from three arrays in **Fig. 2** and sample 1 in **Fig. 4** in the main text. The intensity traces of all particles are included in **Fig. S3.1a**; (a) particle numbers 151 – 175, (b) particles 176 – 200 and (c) particles 201 – 225. The particles are imaged prior to reaction conditions to enable the grain morphology characterization. Generally, most single crystal traces start decreasing later and at a slower rate than polycrystal traces.*

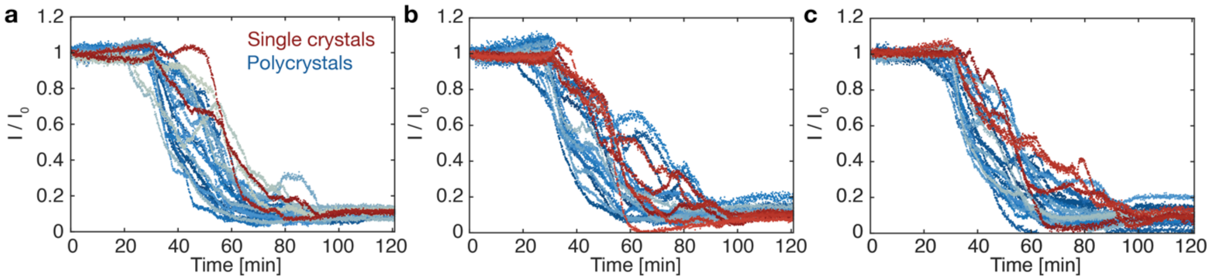

Figure S3.3: *Normalized scattering intensity time traces of 75 particles from three arrays in sample 2 in* ***Fig. 4****, similar to* ***Fig. S3.2****. All intensity traces are included in* ***Fig. S3.1b****; (a) particle numbers 151 – 175, (b) numbers 176 – 200 and (c) numbers 201 – 225. The particles were imaged prior to the reaction conditions for grain morphology characterization.*

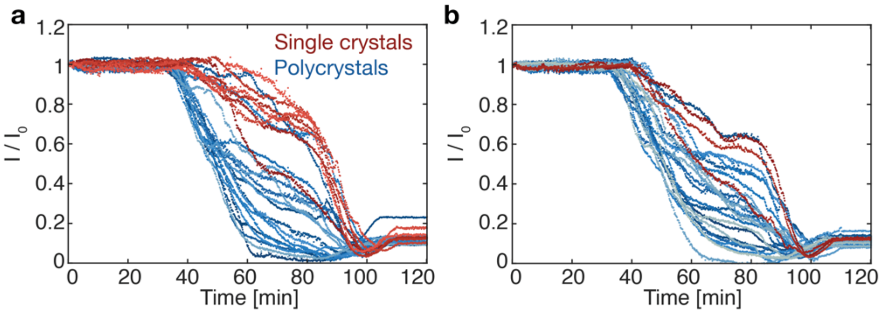

Figure S3.4: *Normalized scattering intensity time traces of 50 particles from two arrays in sample 3 in* ***Fig. 4****, similar to* ***Fig. S3.2.*** *All intensity traces are included in* ***Fig. S3.1c****; (a) particle numbers 151 – 175 and (b) numbers 201 – 225. The particles were imaged prior to the reaction conditions for grain morphology characterization.*

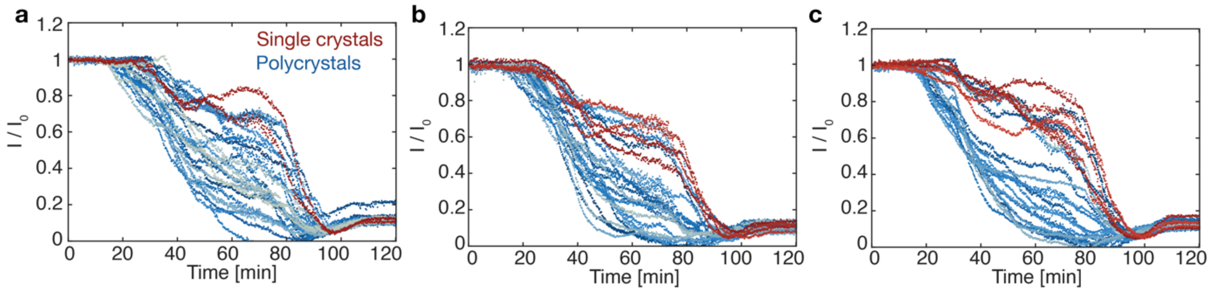

Figure S3.5: *Normalized scattering intensity time traces of 75 particles, each from three arrays in sample 4 in* ***Fig. 4****, similar to* ***Fig. S3.2****. All intensity traces are included in* ***Fig. S3.1d****; (a) particle numbers 151 – 175, (b) numbers 176 – 200 and (c) numbers 201 – 225. The particles were imaged prior to the reaction conditions for grain morphology characterization.*

The limit of 20 % intensity decrease was chosen such that other fluctuations that appeared in the signal, either from fluctuations in the light source, from imperfections in the stabilization of the images, or from dust particles on the sample that could give an uneven background and therefore are hard to properly remove. Hence, to be sure not to count any such fluctuations that are inherent in single particle measurements, we chose 20 % to exclude those other small

decreases in scattering intensity due to other factors than particle oxidation. We show data from such a measurement that had a large dust particle, with the consequence that the background scattering could only be accurately corrected in some particles, in **Fig. S3.6**. These data are not included in any of the figures in the main text.

.

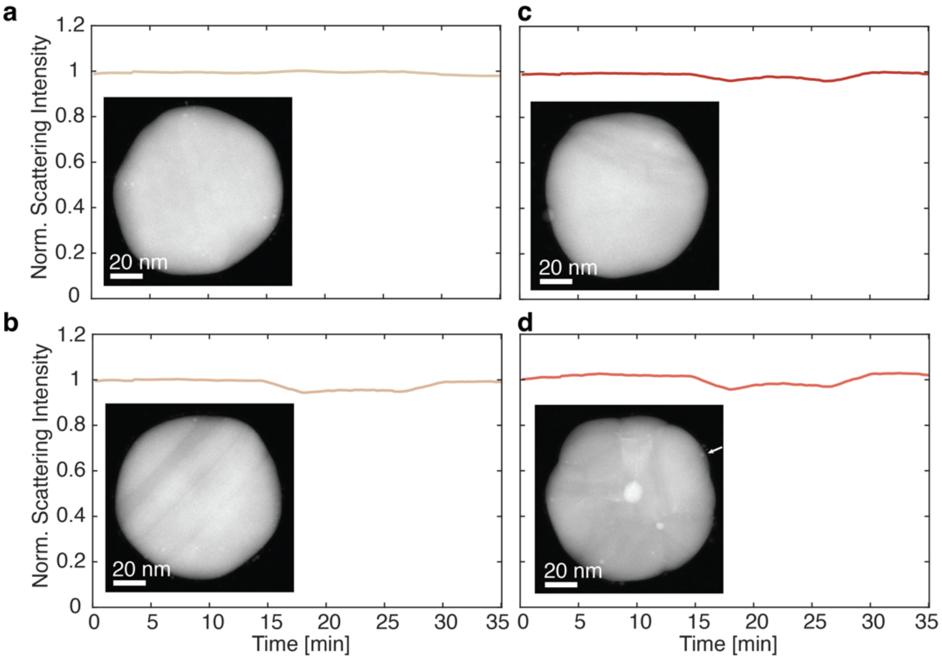

Figure S3.6: *The normalized scattering intensity from four single particles on different positions in the array acquired during 35 minutes of exposure to reaction conditions, after which the reaction was interrupted, and the particles were imaged by ADF-STEM (insets). (a) The background light scattering is properly corrected for and no decrease in the signal is seen, in good agreement with the ADF-STEM image that does not show any signs of oxidation. (b-d) The same artefactual correlated decrease in light scattering intensity is seen in the signals from all three particles at about 13 min of exposure. It originates from light scattered by a nearby dust particle that convolutes the Au particle reference signal. Note that also these three particles don't show sizeable oxidation (a tiny, oxidized region in (d) is indicated by the arrow) in the ADF-STEM images, corroborating that the decrease in light scattering intensity originates from the background scattering of the sample.*

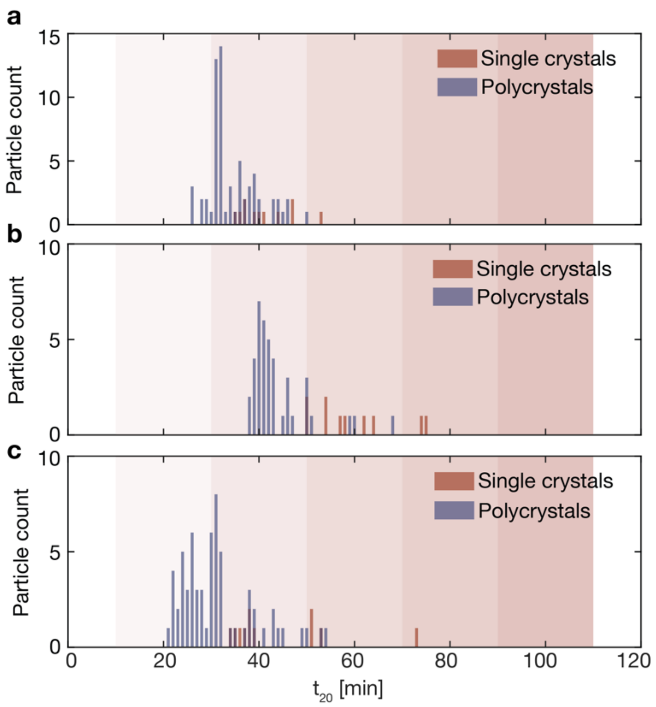

Figure S3.7: *The t_{20} distributions from three additional samples to the one measured in* ***Fig. 2c*** *in the main text, the particles are grouped into single crystals (red) and polycrystals (blue). The red fields mark the O_2 gas concentration during the reaction; 0.04, 0.08, 0.12, 0.16, and 0.2 %. The mean and standard deviation for single crystals and polycrystals from each sample are (a) from 75 particles from sample 2 (cf. Fig. S3.1): $\bar{t}_{20,single}$ = 42 ± 5.7 min and $\bar{t}_{20,poly}$ = 35 ± 5.3 min, (b) from 50 particles from sample 3: $\bar{t}_{20,single}$ = 60 ± 8.9 min and $\bar{t}_{20,poly}$ = 44 ± 6.4 min, and (c) from 75 particles from sample 4: $\bar{t}_{20,single}$ = 45 ± 12 min and $\bar{t}_{20,poly}$ = 32 ± 7.9 min.*

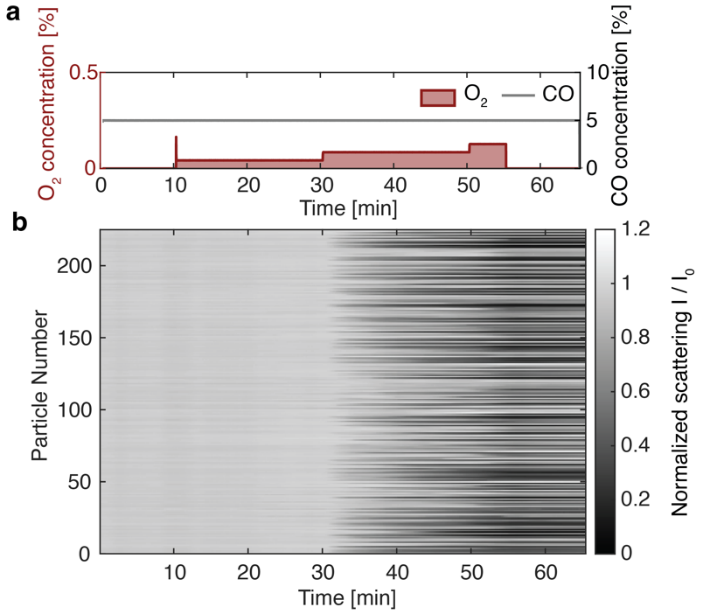

Figure S3.8: *(a) The concentrations of O_2 and CO in the gas stream in the experiment depicted in **Fig. 3** in the main text (same figure as in **Fig. 3**). (b) The normalized scattering intensity of the 225 Cu particles in the sample of which the intensities of 4 particles and their TEM images from before and STEM from after the terminated reaction sequence are depicted in **Fig. 3**.*

S4: Enhanced oxidation along grain boundaries

We measured the maximum oxide depth, i.e., the depth where the oxide had grown the deepest into the Cu lattice, per particle from ADF-STEM images. The maximum depth coincides in most polycrystals with the oxide apex and is found along the grain boundary (**Fig. S4.1a**). In the single crystals the maximum depth is normally at the center of a straight oxide front (**Fig. S4.1b**). Plotting the maximum oxide depth as a function of the volume oxidation fraction from three samples (**Fig. S4.1c**) we can make two observations. First, many of the polycrystals have a deeper oxide and a higher oxidation fraction compared to the single crystals in the same samples. Second, most of the single crystals lie beneath the black line that indicates the oxide depth obtained from *one* linear oxide front. In contrast, many of the polycrystals are found above this line, which indicates that they have an apexed oxide shape at the same oxidation fraction, which is confirmed from the ADF-STEM images. Several polycrystals are also found below the one-oxide-front-line, which is natural in cases when the oxide starts to grow at several sites simultaneously, since thereby the oxide depth will be less at each of the sites for the same particle volume oxidation fraction. Hence, the maximum oxide depth will be smaller.

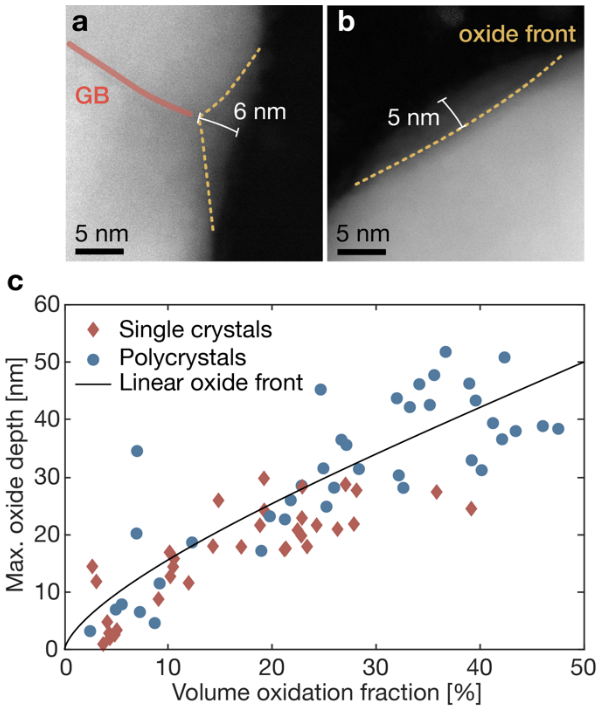

Figure S4.1: *Examples of ADF-STEM images of (a) apexed oxide growth at a grain boundary and (b) linear oxide growth. The interface between the growing oxide and the metal is marked by the dashed yellow line and the grain boundary in (a) by a red line. At the grain boundary, the oxide has grown deeper, which leads to the formation of an apex. In contrast in (b) where grain boundaries are lacking the oxide – metal interface is straight. The maximum oxide depth is indicated in both (a) and (b) by the white line. (c) The maximum oxide depth vs. the oxidation fraction as obtained from ADF-STEM images from three samples oxidized to different volume fractions. Note the larger max. oxide depth in many of the polycrystals (blue circles) compared to single crystals (red diamonds). The oxide depth from one linear-oxide-front vs. oxidation fraction is indicated by the black line. Note that most of the single crystals lie below the linear-oxide-front-line, which indicates that single crystals do not have the oxide apex, whereas many of the polycrystals have a deeper oxide than expected from one linear front.*

S5: STEM oxide characterization

Imaging particles by ADF-STEM after terminating the reaction sequence (cf. Fig. S1.1c) after 35 min (**Fig. S5.1a)** and after 25 min (**Fig. S5.1b)**, at some positions the subsurface oxide growth is apparent. By high-magnification STEM imaging, lattice fringes are visible at some areas that appear dark due to low electron scattering. These areas are expected to be oxide (marked by arrows), and areas that appear brighter due to higher electron scattering are metal. We calculated the fast Fourier transform (FFT, using Digital Micrograph 3) and identified the diffraction spots in the reciprocal space that corresponded to the areas identified as oxide and metal, respectively. From these diffraction spots we then calculated the lattice constants. This analysis yielded the lattice parameters 2.07 Å (green areas) and 2.47 Å (orange areas) which corresponds to the Cu(111) and Cu_2O(111) lattice orientations, respectively (**Fig. S5.1e**). Similarly, in **Fig. S5.1f** we obtained 1.79 Å (green areas) and 2.12 Å (orange areas) which we interpret as the lattice distance of Cu(200) and Cu_2O(200), respectively. The areas found to be Cu_2O are colored orange and the areas with Cu fringes are colored green in the false-colored images and are overlayed on the original STEM images (**Fig. S5.1c, d**), which is colored blue.

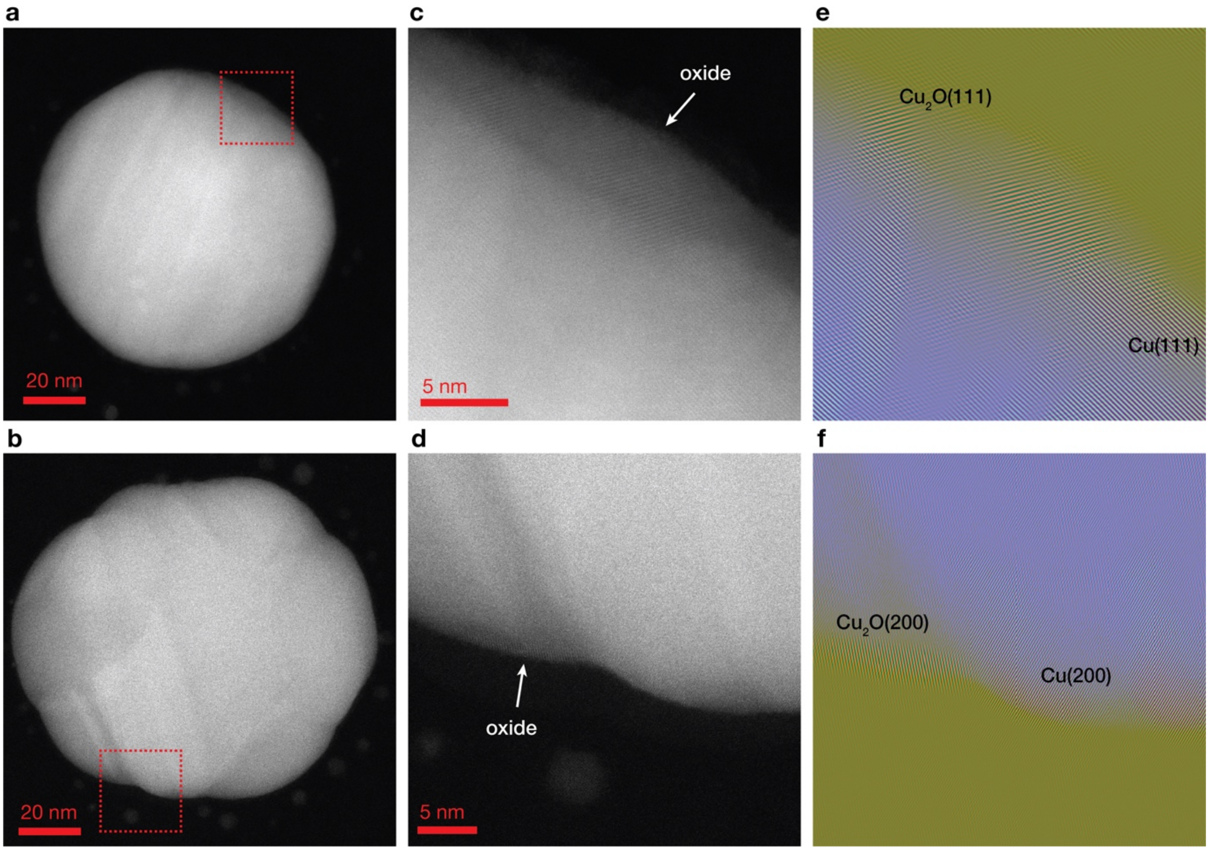

Figure S5.1: *STEM images of two particles, for which the reaction sequence has been interrupted (a) after 35 min (5 min into the 0.08 % O_2 step) and (b) after 25 min (15 min into the 0.04 % O_2 step). The areas for the high-magnification-images (c, d) are marked with the dashed squares. (c, d) Images taken at high magnification, which show lattice fringes at positions in the image where we expect Cu and Cu_2O. (e, f) False colored composite images of the inverse FFT (IFFT) of the diffraction spots from the FFT of (c, d) overlayed onto the images in (c, d), which are colored blue. Areas that correspond to oxide are colored orange and areas that correspond to metallic Cu are green. The distance between diffraction spots is measured to give lattice constants corresponding to lattice orientations indicated in the images.*

S6: Mass spectrometry of the CO oxidation reaction over large-area samples

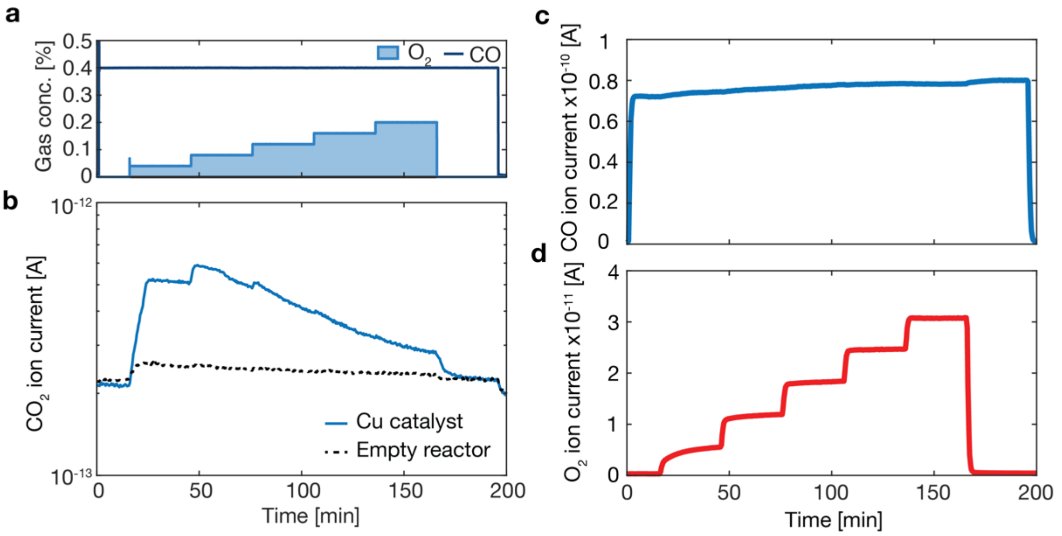

Figure S6.1: *(a) Schematic of the used reactant concentrations, i.e., constant 0.4 % CO and increasing O_2 from 0 % to 0.2 % for the pocket reactor [2] experiments on Cu nanoparticle ensemble samples. (b) The CO_2 reaction product ion current measured for a 6.3 x 10.5 mm sample covered by a quasi-random array of Cu nanodisks with 140 nm diameter, nanofabricated by hole mask colloidal lithography[3] onto an oxidized silicon substrate (blue) plotted together with the response from an empty reactor (dashed curve). The reactant ion currents: (c) CO and (d) O_2 measured simultaneously confirm that some CO and O_2 is consumed in the reaction. Specifically, O_2 consumption is seen in that the first O_2 step is rounded. Furthermore, we see an increase of the CO baseline, which is the consequence of less CO being oxidized due to increased surface oxidation (and thus lower activity) of the Cu particles as the O_2 concentration is increased. The particles are on a flat substrate, which means there is excess gas volume in the reactor and therefore a large fraction of the gases fed into the pocket reactor has not reacted, i.e., conversion is low.*

S7: Varying the CO background concentration

We repeated the CO oxidation reaction experiment on four samples that were all pretreated in the same way, namely at 400 °C for 2 hours, and varied the background CO concentration to see if this influenced the critical O_2 exposure for Cu oxidation onset. Hence, all these samples have about the same initial grain boundary density distribution. Interestingly, we don't observe a clear dependence of the critical O_2 exposure on the CO concentration. The Cu surface is pre-covered with CO, hence, for Cu oxidation to start CO needs to desorb, or oxidize and desorb as CO_2. Therefore, if CO desorption alone controls the Cu oxidation onset, it would be seen in that the critical O_2 exposure would depend on the CO background concentration. No dependence on the CO concentration is observed in samples pre-treated at 400 °C, and we conclude that CO oxidation further delays the Cu oxidation after initial CO desorption.

However, in **Fig. 7e** in the main text, we are comparing samples with different grain boundary distributions and therefore, observe a dependence of the average CO desorption on the pretreatment temperature of each sample.

A second aspect one can note from these experiments is that in the low CO concentration experiments, the difference between the single and polycrystal critical O_2 exposure has decreased. We are speculating that it is because at lower CO coverage on the surface, the CO oxidation on the facets of the single crystals becomes less competitive to Cu oxidation. Hence, the difference between single and polycrystals is reduced.

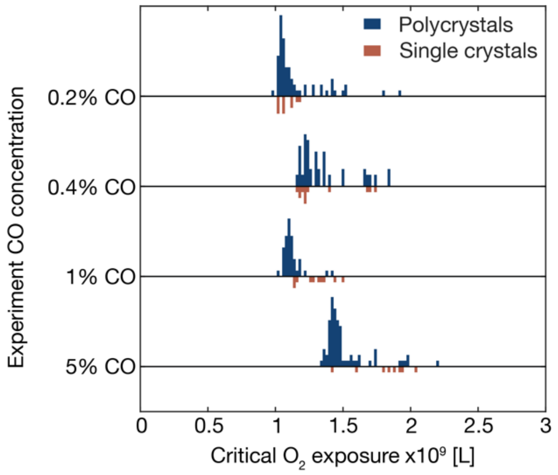

Figure S7.1: *The critical O_2 exposure for Cu oxidation onset (similar to* ***Fig. 4c*** *in the main text) determined for 0.2, 0.4, 1 and 5 % CO concentration in the gas feed. There is no clear dependence of the critical O_2 exposure on the CO concentration in the investigated range.*

S8: Oxide nucleation positions and delayed Kirkendall void formation

We analyzed example particles from two samples by imaging them by STEM after 35 min exposure to reaction conditions up until 0.08 % O_2 (sequence displayed in **Fig. S1.1c**). We observe more oxide nucleation positions on the polycrystals (**Fig. S8.1b** and **S8.2b**) compared to the single crystals (**Fig. S8.1a** and **S8.2a**).

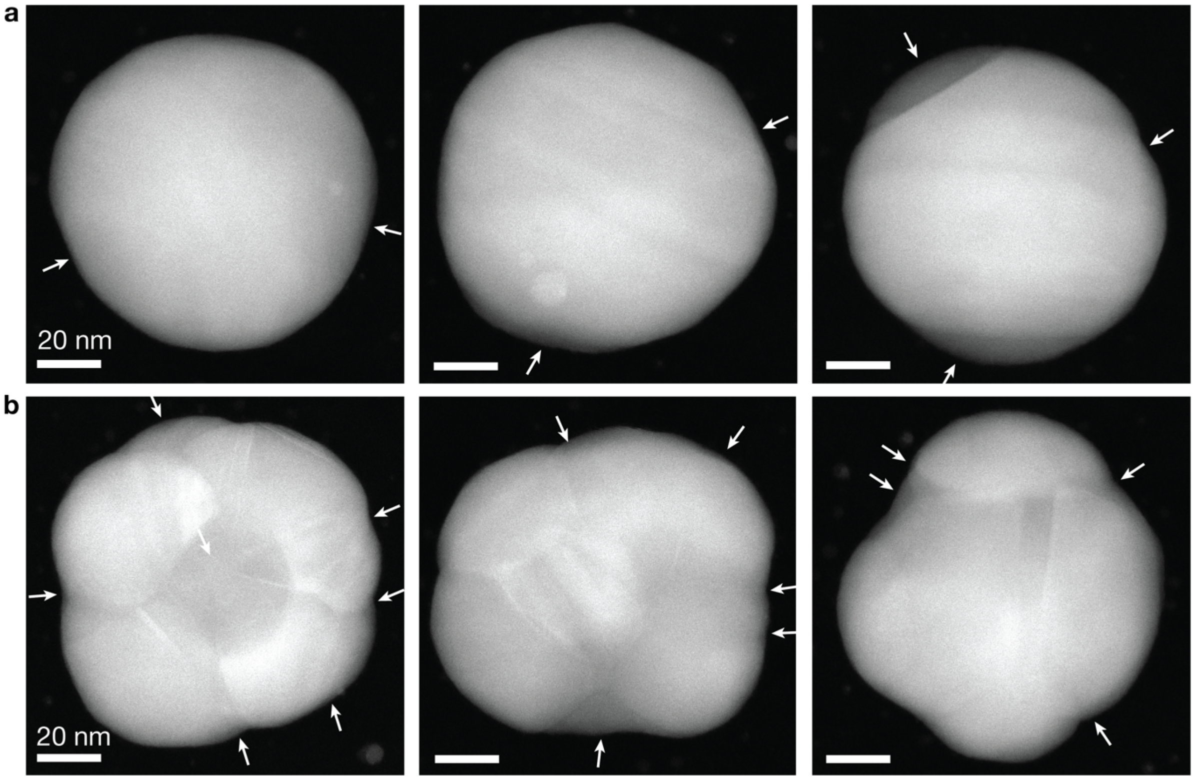

Figure S8.1: *Examples of STEM images of a random selection of particles taken after 35 minutes at reaction conditions up to 0.08 %* O_2*. (a) 3 single crystals show sizable oxidation at 2 – 3 positions marked by the arrows. (b) 3 polycrystals with distinct oxide growth at 4 – 7 positions marked by the arrows.*

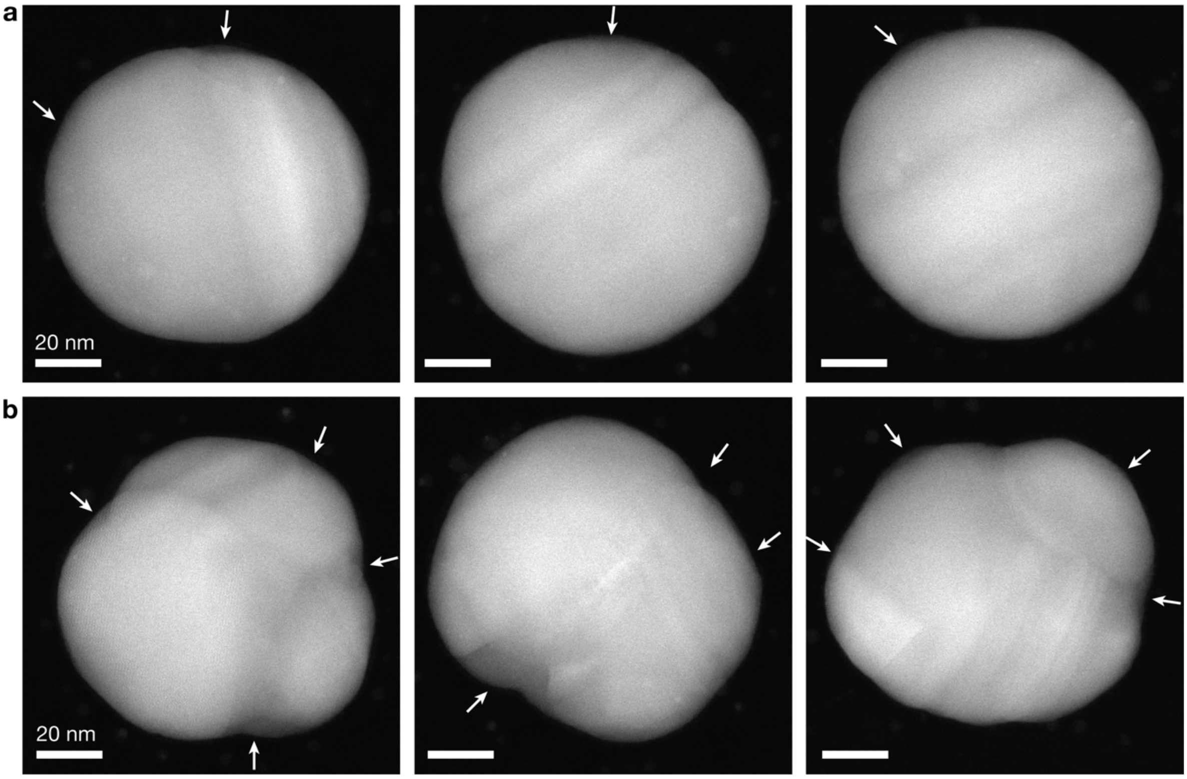

Figure S8.2: *Examples of STEM images of a random selection of particles from another sample treated the same way as in* ***Fig. S8.1*** *up to 0.08 % O_2 after a total of 35 minutes in reaction conditions. (a) 3 single crystals showing sizeable oxidation at 1 – 2 positions marked by the arrows. (b) 3 polycrystals with oxide growth at 3 – 4 positions marked by the arrows.*

We also show some particles imaged after 25 minutes under reaction conditions, with an O_2 concentration of 0.04 %. No oxidation is observed in the single crystals (**Fig. S8.3**) whereas, some, but clearly not all, of the polycrystals exhibit sizeable oxidation (**Fig. S8.4**).

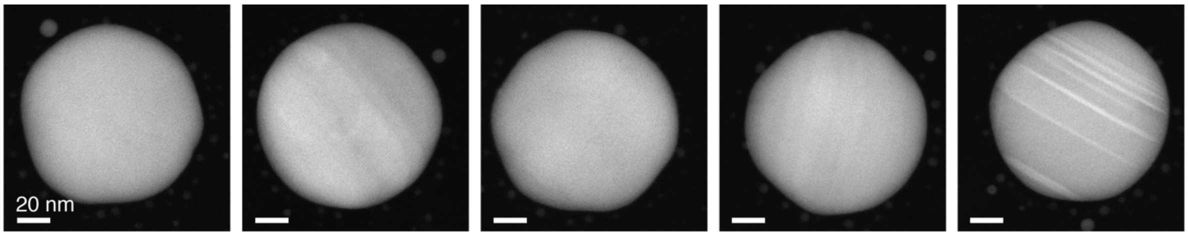

Figure S8.3: *Examples of STEM images of a random selection of single particles treated for 25 minutes at reaction conditions up to 0.04 % O_2. No sizeable oxidation is observed. All scalebars are 20 nm.*

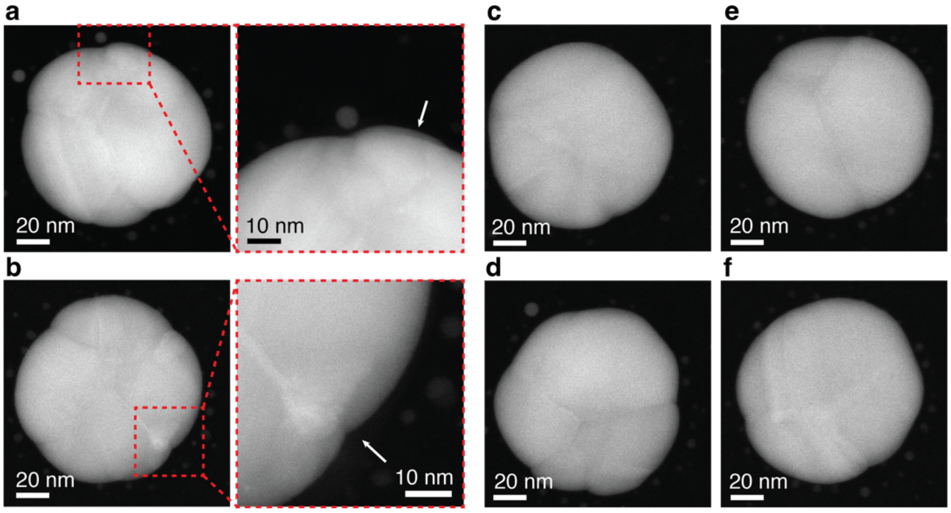

Figure S8.4: *Examples of STEM images of a random selection of polycrystalline particles from the same sample as in* ***Fig. S8.3****, treated for 25 minutes at reaction conditions up to 0.04 %* O_2*. Two particles (a, b) show sizeable oxidation at two positions for which zoom-in images are provided in which the position of oxide growth is marked by the arrow. In (c-f) we show examples of 4 polycrystals that do not yet show sizeable oxidation after 25 minutes under reaction conditions. All scalebar lengths are marked in the figure.*

As a second aspect, analyzing 29 particles imaged by STEM from the sample discussed in **Fig. 3** in the main text (4 of the particles are also shown in **Fig. 3** in the main text), we see that none of the particles has formed a Kirkendall void (**Fig. S8.5-6**). This, despite volume oxidation fractions of the polycrystals between 19 – 33 %, and of the single crystals between 2 – 23 %. This sample was exposed to the reaction conditions for 55 minutes along the reaction sequence depicted in **Fig. S1.1c**, which means it was in reaction conditions up to the step of 0.12 % O_2.

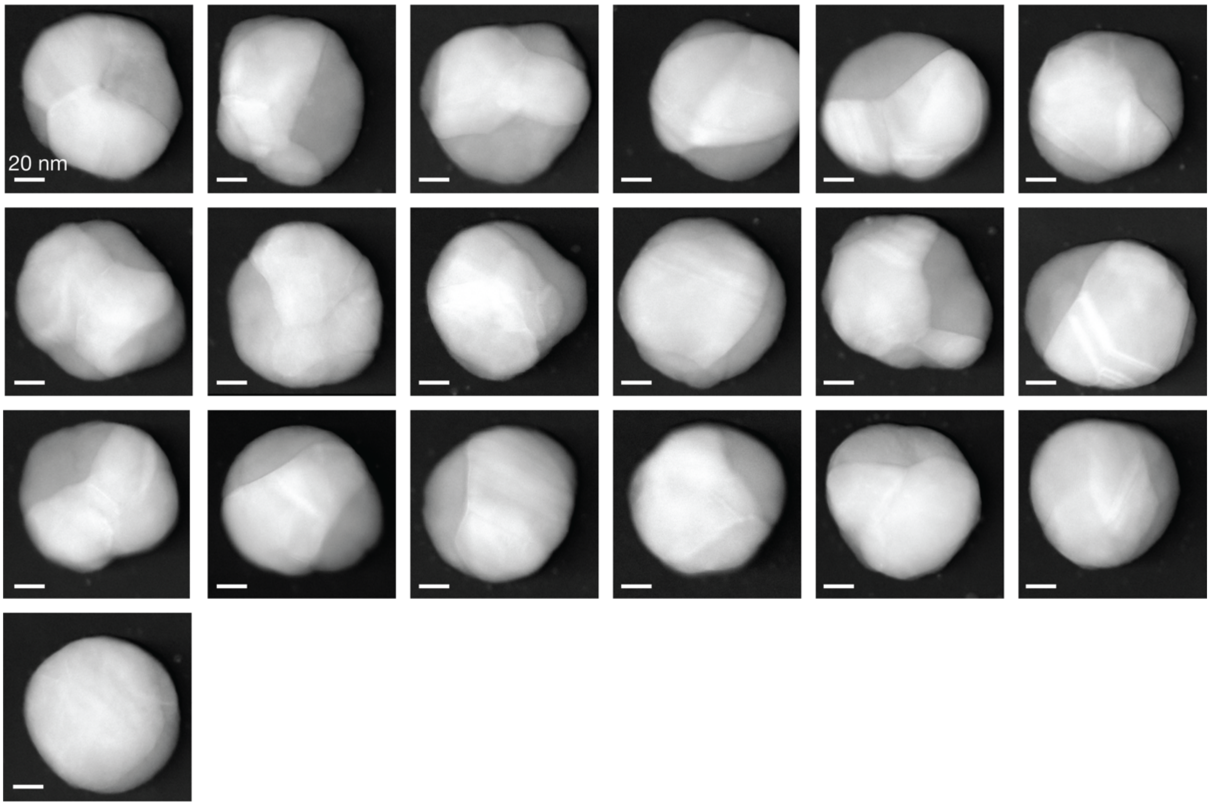

Figure S8.5: *Examples of STEM images of polycrystals on the same sample as the particles in **Fig. 3** in the main text (the first two particles are the same as in **Fig. 3**). In none of the particles has a Kirkendall void has formed.*

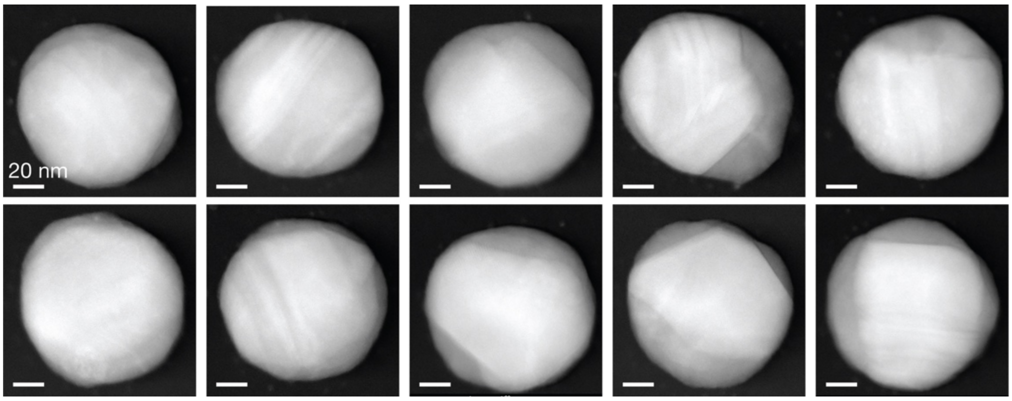

Figure S8.6: *Examples of STEM images of single crystals on the same sample as the particles in **Fig. 3** in the main text (the first two particles are the same as in **Fig. 3**). Also in the single crystals, no Kirkendall voids are seen.*

S9: Control experiment without CO

A control experiment was performed where the O_2 concentration was increased in the same way as described in **Fig. 1a** and **Fig. S1.1**, in steps of 0.04 percentage but without CO present in the feed (**Fig. S9.1a**). In these conditions, the particles start to form the oxide already upon exposure to the lowest O_2 concentration of 0.04 % (**Fig. S9.1b, c**). A similar experiment as in **Fig. S9.1a** but interrupted after 50 minutes was also performed to enable *ex situ* ADF-STEM imaging after the experiment (**Fig. S9.2a**). The selection of images of particles oxidized in absence of CO shows that the oxide has started to grow at a handful of sites around the particle. This is still a smaller number of sites compared to similar Cu particles oxidized at higher O_2 partial pressures[4–6], but significantly more than for a sample whose O_2 exposure also was interrupted after 50 minutes but in 5 % CO (**Fig. S9.2b**). Another difference we note is that the oxide appears to be more porous when grown in pure O_2, which might be a sign that the oxide is nucleated at many positions, which inhibits a uniform oxide lattice orientation. In contrast, for the particles oxidized under CO + O_2, the oxide surface appears smoother, and when analyzing the oxide that was formed in CO + O_2, but in lower O_2 concentrations, we were able to identify one single lattice orientation (see **Section S5** above).

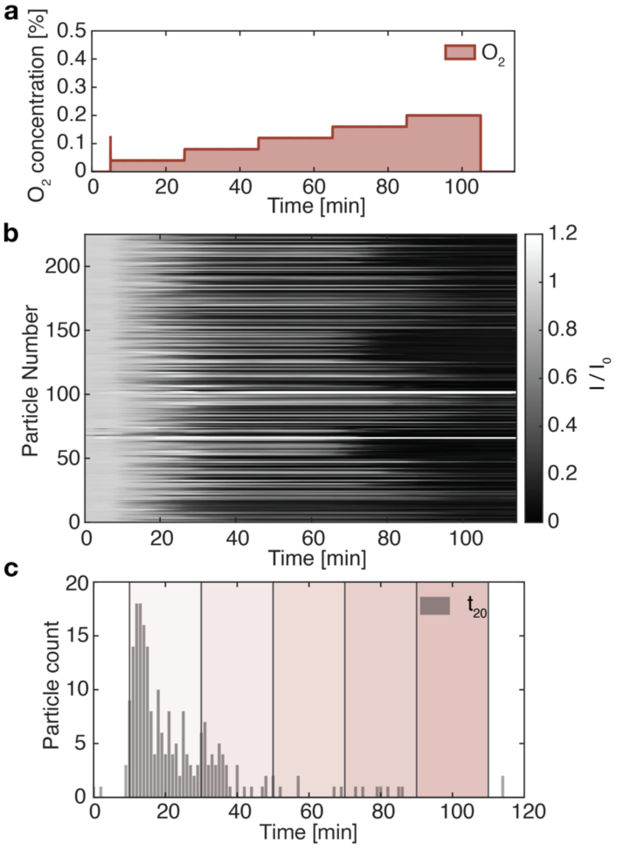

Figure S9.1: *(a) A control experiment was run at 250 °C with identical O_2 concentration increase as in* ***Fig. 1a*** *and* ***Fig. S1.1*** *but without CO present. (b) The correspondingly obtained normalized scattering intensities of the 225 particles in the sample reveal that most particles decrease in intensity already when O_2 is first introduced after 5 min at a concentration of 0.04 %. (c) The t_{20} histogram of all the particles in (b), showing clearly that the oxidation starts during the first exposure to O_2 at a concentration of 0.04 %.*

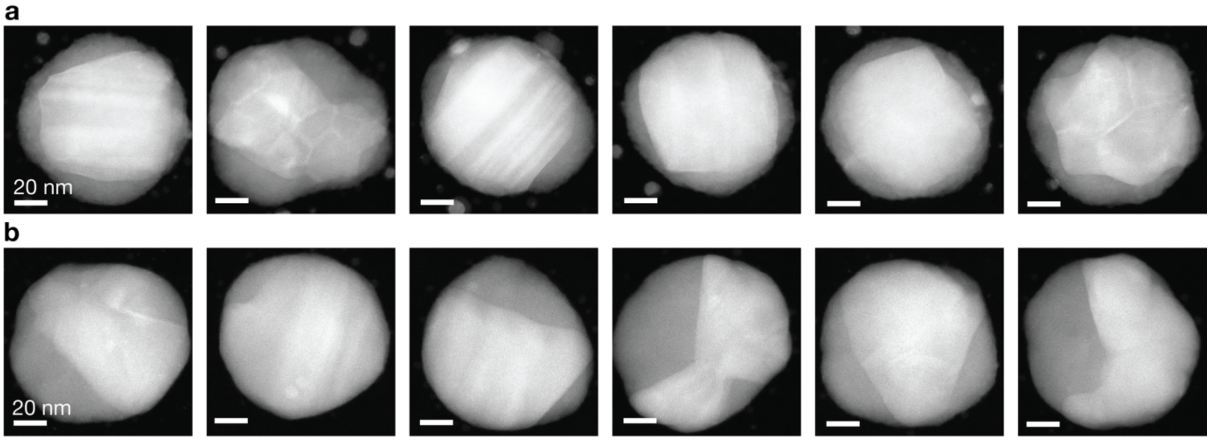

Figure S9.2: *Examples of STEM images of a random selection of particles from (a) a similar experiment to that described in **Fig. S9.1** where the experiment was interrupted for ex situ STEM imaging after 50 minutes of exposure to pure* O_2 *in increasing concentration steps. (b) Same as in (a) but in a 5 % CO background.*

S10: Density functional theory calculations

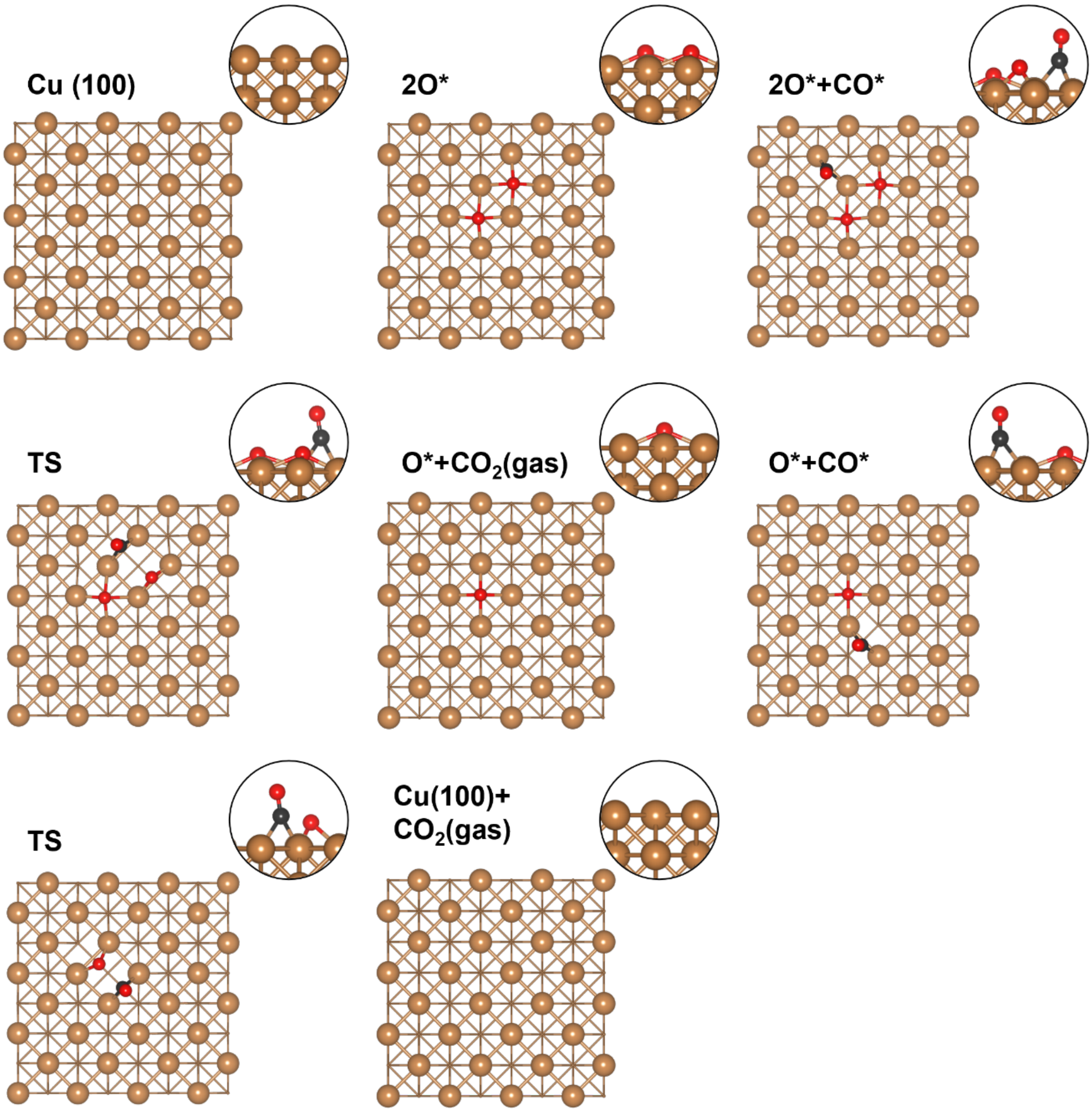

Figure S10.1: *Detailed top and side views (in circles) for the CO oxidation reaction on the Cu(100) surface. The structures correspond to the different steps presented in the reaction diagram in **Fig. 5** of the main text.*

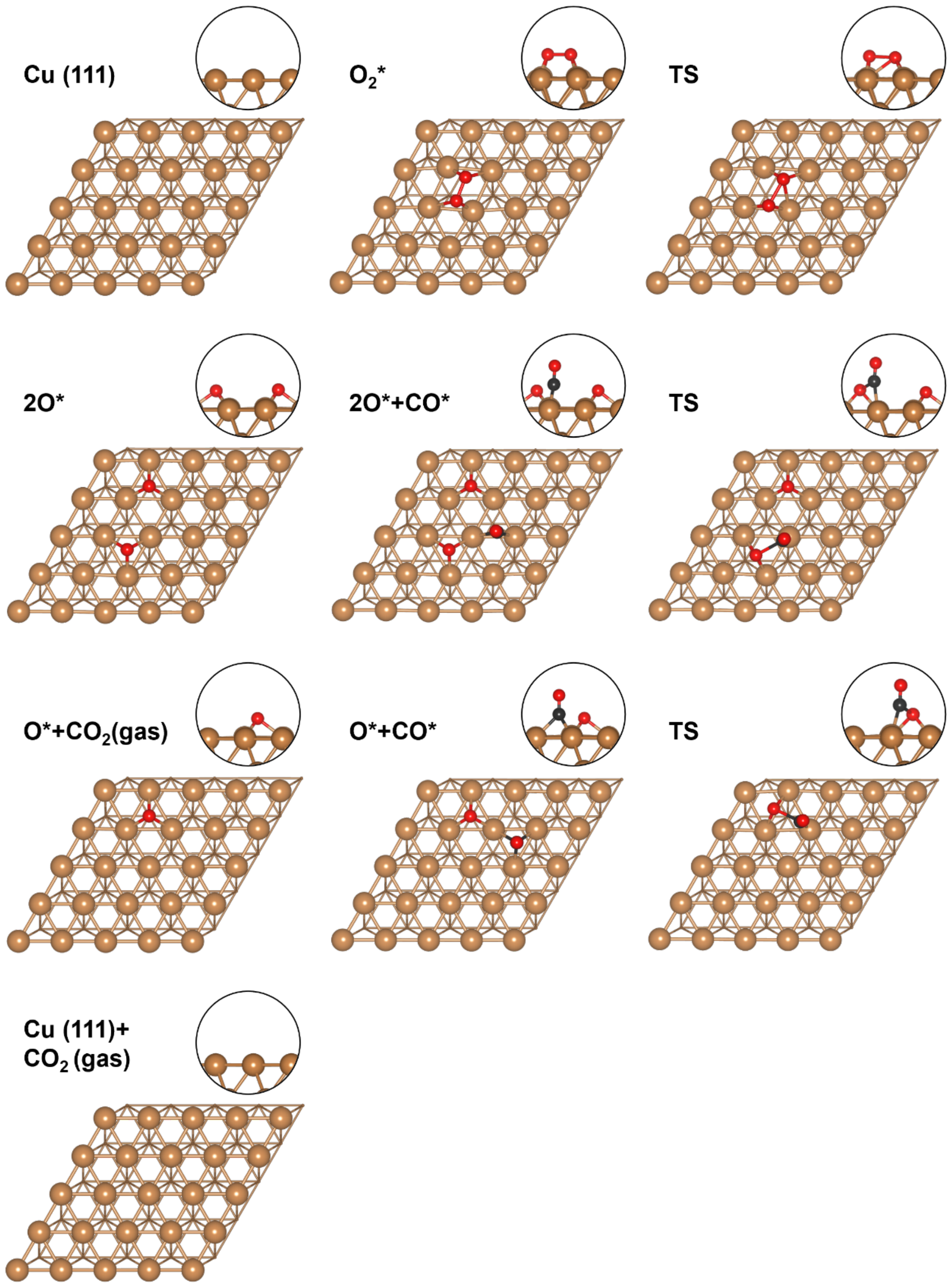

Figure S10.2: *Detailed top and side views (in circles) for the CO oxidation reaction on the Cu(111) surface. The structures correspond to the different reaction steps presented in the reaction diagram in* ***Fig. 5*** *of the main text.*

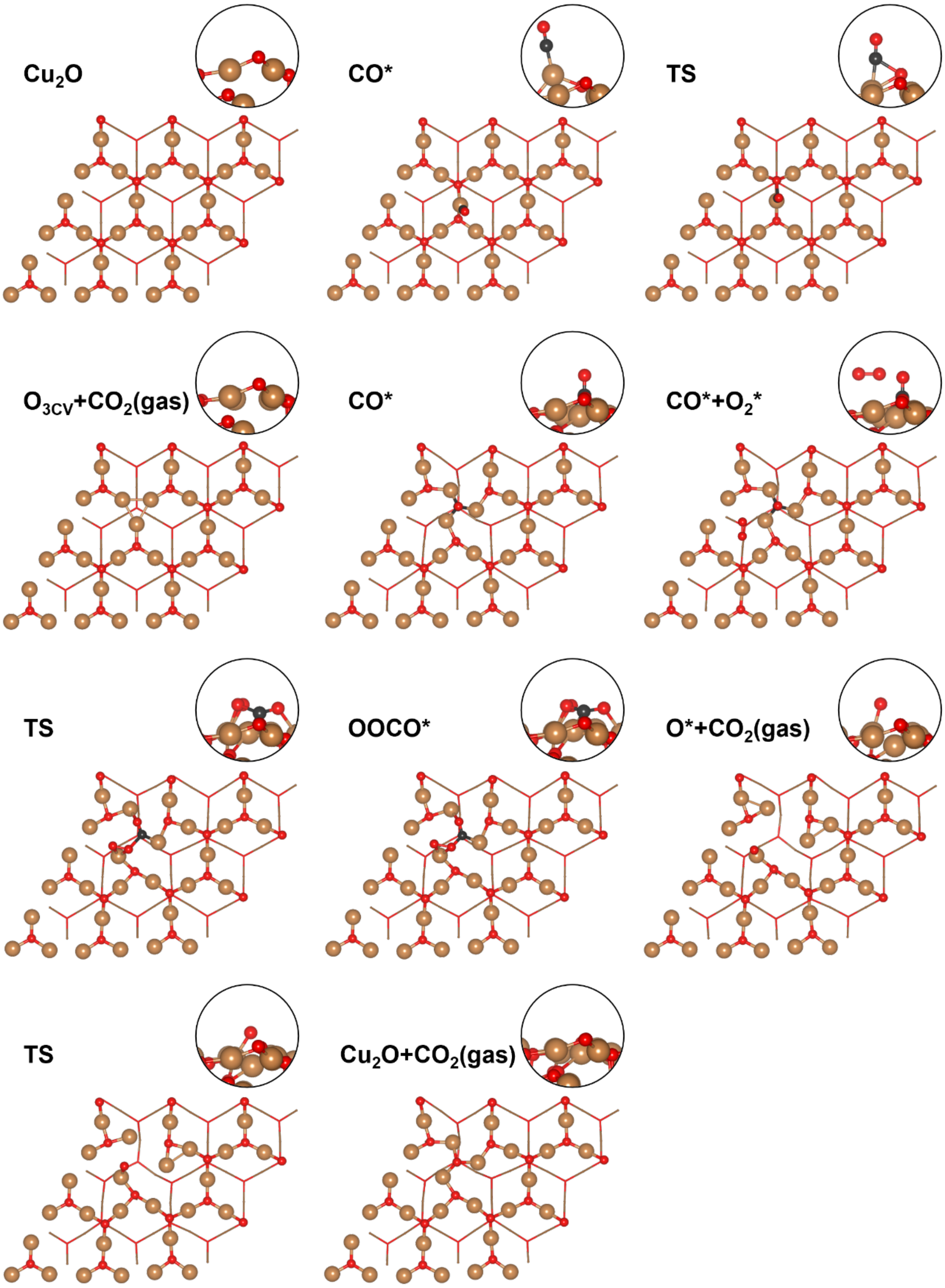

Figure S10.3: *Detailed top and side views (in circles) for the CO oxidation reaction on the (111) O-terminated (1x1)* V_{CuCUS} Cu_2O *surface. The structures correspond to the different steps presented in the reaction diagram in* ***Fig. 5*** *in the main text.*

S10.1 Comparisons between DFT and DFT+U

As mentioned in the manuscript, the total reaction energy ($\Delta E_{reaction} = 2E_{CO2} - 2E_{CO} - E_{O2}$) of the oxidized system is different to that of the metallic systems. This mismatch is the result of the application of the Hubbard U correction to both Cu's d and O's p orbitals in Cu_2O, which caused a change in the reference energy values for CO, O_2 and CO_2. Despite the changes in energies, no appreciable changes in bond lengths were observed, as shown in **Table S10.1**.

Table S10.1: *Bond lengths and energy comparisons for the CO, O_2 and CO_2 references.*

Adsorbate	Bond length DFT (Å)	Bond length DFT+U (Å)	$E_{DFT+U} - E_{DFT}$ (eV)
CO	1.136	1.139	1.48
CO_2	1.176	1.178	2.69
O_2	1.232	1.212	1.65

Furthermore, to verify that the predicted energy trends hold regardless of the computational method, single point energies were computed using just DFT on the DFT+U optimized surfaces. A comparison between the DFT and DFT+U calculated energies is provided in **Fig. S10.4**. The steps leading to the formation of the oxygen vacancy (CO adsorption and oxidation) and the final state appear to be the only steps with different energies between the two methods, the remainder of the reaction steps almost perfectly overlap. More importantly, the total reaction energy paths have similar values, which remain higher than those found on the metallic systems, which were identified as more active for the CO oxidation reaction.

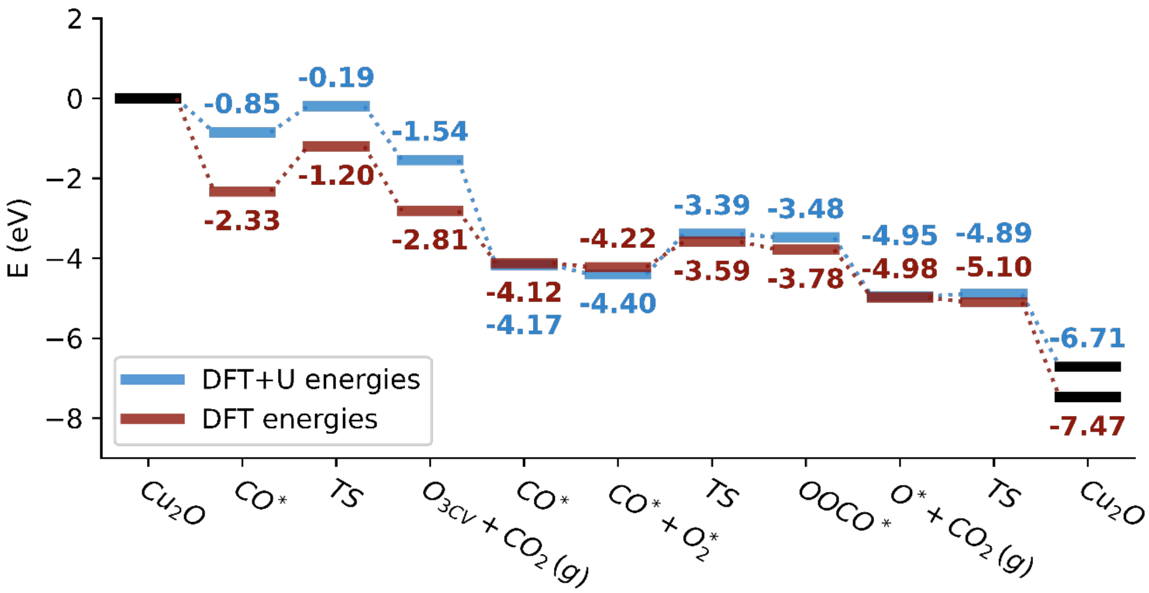

Figure S10.4: *Comparison of DFT and DFT+U predicted reaction energies for the CO oxidation reaction on the (111) O-terminated (1x1) V_{CuCUS} Cu_2O surface.*

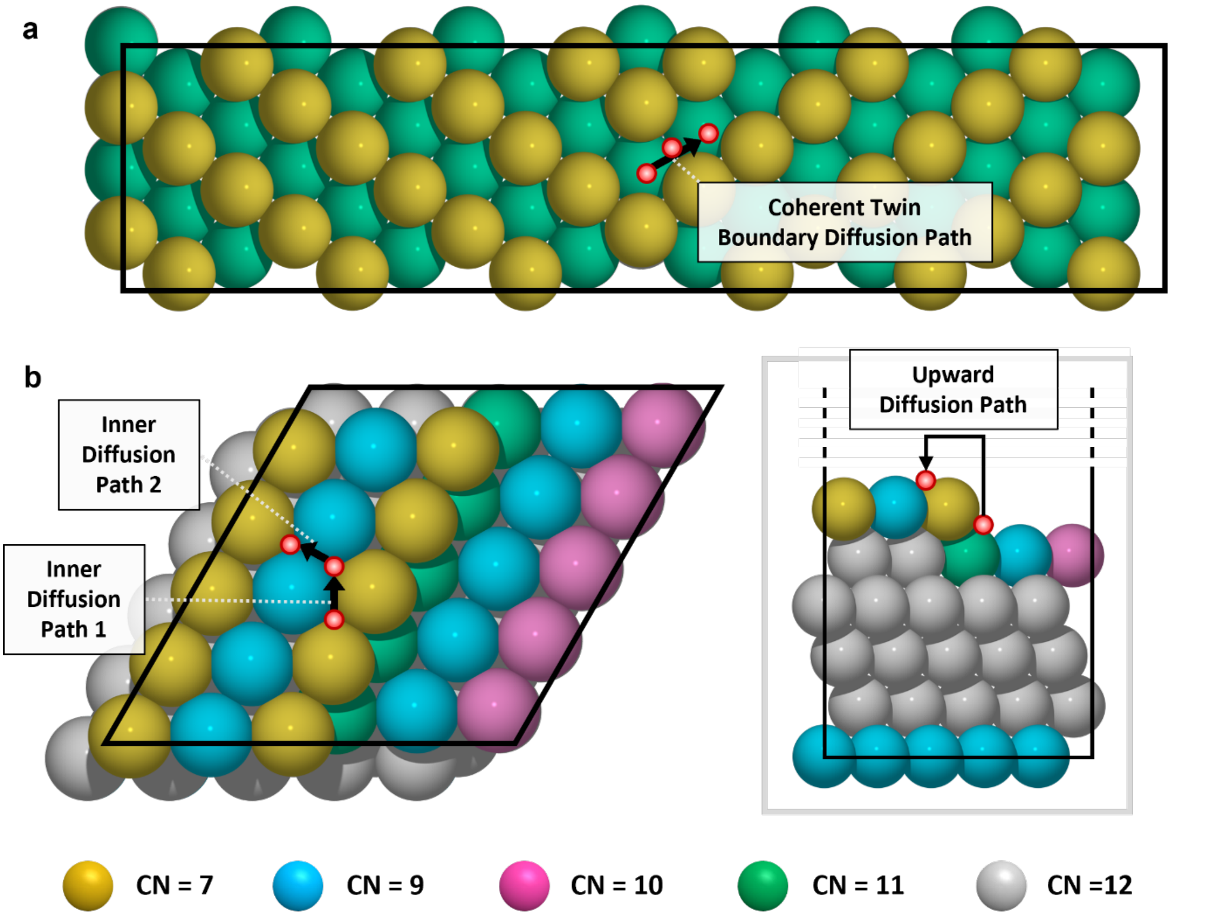

Figure S10.5: *Coordination environments and considered O diffusion paths on (a) Cu's coherent twin boundary, (b) the (111) with {111} microfacet step-edge (on the right: side view). CN stands for coordination number.*

S10.2 Summary Infographic

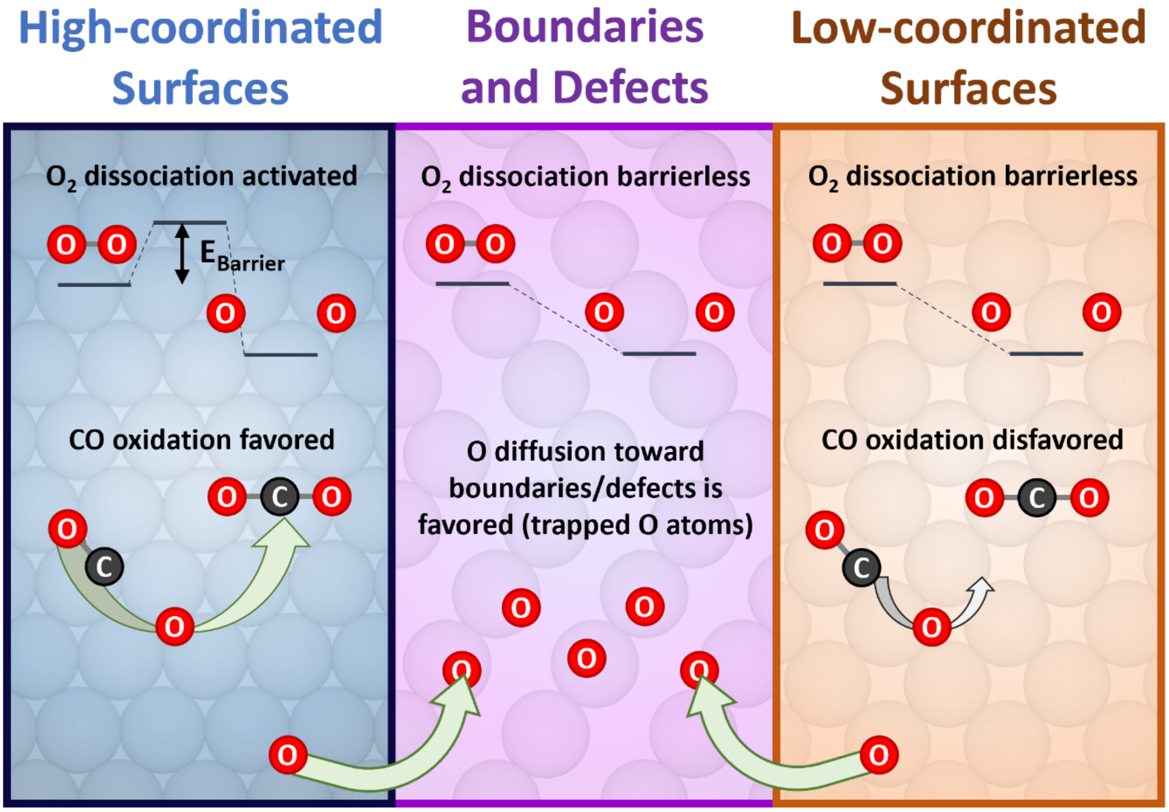

Figure S10.6: *Summary infographic for the computational results observed for all surfaces that include high-coordinated surfaces, such as Cu (111), low-coordinated surfaces, such as Cu(100), and grain boundaries or lesser coordinated surfaces, such as the studied coherent twin boundary or the (111) step-edge.*

S10.3 Details on Computational methods

Table S10.2. *Computed vs reported geometric parameters.*

System	Space Group	Bravais Lattice	Computed Lattice Constants (Å)	Experimental Lattice Constants (Å)
Cu[7]	Fm3m	Cubic	3.626	3.615
Cu_2O[8]	Pn3m	Cubic	4.256	4.269

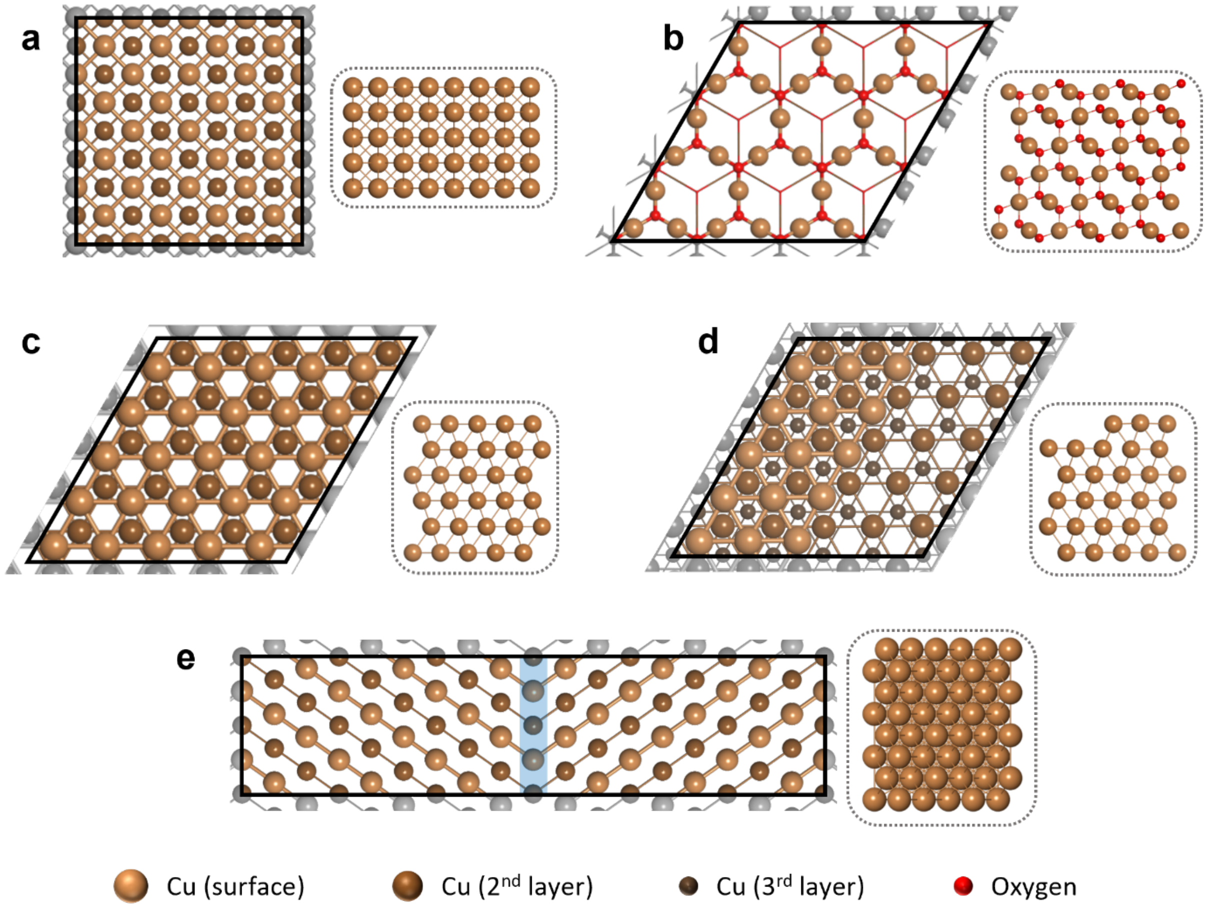

Figure S10.7: *Top and side (circled) views of the used slab models, corresponding to (a) Cu(100), (b) non-stoichiometric* $Cu_2O(111)$ *surface, (c) Cu(111), (d) step-edge Cu(111), and (e) Cu coherent twin boundary (twin boundary highlighted in blue).*

References

1. Nilsson, S., Nielsen, M. R., Fritzsche, J., Langhammer, C. & Kadkhodazadeh, S. Competing oxidation mechanisms in Cu nanoparticles and their plasmonic signatures. *Nanoscale* **14**, (2022).
2. Tiburski, C. *et al.* Light-Off in Plasmon-Mediated Photocatalysis. *ACS Nano* **15**, 11535–11542 (2021).
3. Fredriksson, H. *et al.* Hole–mask colloidal lithography. *Advanced Materials* **19**, 4297–4302 (2007).
4. Nilsson, S., Albinsson, D., Antosiewicz, T. J., Fritzsche, J. & Langhammer, C. Resolving single Cu nanoparticle oxidation and Kirkendall void formation with in situ plasmonic nanospectroscopy and electrodynamic simulations. *Nanoscale* **11**, 20725–20733 (2019).
5. Rice, K. P., Paterson, A. S. & Stoykovich, M. P. Nanoscale Kirkendall effect and oxidation kinetics in copper nanocrystals characterized by real-time, in situ optical spectroscopy. *Particle & Particle Systems Characterization* **32**, 373–380 (2015).
6. Susman, M. D., Feldman, Y., Bendikov, T. A., Vaskevich, A. & Rubinstein, I. Real-time plasmon spectroscopy study of the solid-state oxidation and Kirkendall void formation in copper nanoparticles. *Nanoscale* **9**, 12573–12589 (2017).
7. Rumble, J., Lide David & Bruno Thomas. *CRC handbook of chemistry and physics : a ready-reference book of chemical and physical data*. (2018).
8. Kirfel, A. & Eichhorn, K. Accurate structure analysis with synchrotron radiation. The electron density in Al2O3 and Cu2O. *Acta Crystallographica Section A* **46**, 271–284 (1990).
